# Toward Intelligent Chemical Mechanical Polishing: Integrating Multiscale Modeling and Machine Learning

**DOI:** 10.3390/ma19194205

**Published:** 2026-10-02

**Authors:** Xin Wu, Quanzhou Yao

**Affiliations:** School of Automation and Intelligent Manufacturing, Southern University of Science and Technology, Shenzhen 518055, China

**Keywords:** chemical mechanical polishing, material removal mechanism, multiscale modeling, machine learning, process control, sustainability

## Abstract

Chemical mechanical polishing (CMP) remains the cornerstone planarization technology in advanced semiconductor manufacturing. As device architectures transition toward FinFETs, gate-all-around structures, heterogeneous integration, and wide-bandgap semiconductors, CMP must simultaneously deliver angstrom-level flatness, high selectivity, low damage, and improved sustainability. Against this background, research in CMP is moving beyond empirical trial-and-error toward a more integrated paradigm that combines consumables design, physics-based modeling, and data-driven intelligence. This review systematically summarizes recent progress in CMP from four interrelated perspectives. First, we examine advances in slurry and abrasive design, including morphology engineering, porous and core–shell abrasives, defect-controlled ceria systems, and environmentally friendlier chemical formulations, together with the emergence of photo-, electro-, ultrasonic-, plasma-, and gas-assisted CMP. Second, we review the multiscale modeling landscape, spanning macroscale planarization and profile evolution, mesoscale contact mechanics and slurry transport, and atomistic simulations based on first-principles calculations and reactive molecular dynamics. Third, we assess the rapidly growing role of machine learning in CMP, with emphasis on material removal rate prediction, surface quality evaluation, process monitoring, virtual metrology, and intelligent run-to-run control. Fourth, we discuss sustainability-oriented CMP, including source reduction, wastewater treatment and recycling, and the use of modeling and digital control to reduce non-productive resource consumption. By synthesizing developments across these areas, this manuscript highlights the current gaps in cross-scale model integration, physics-informed intelligent control, transferability across tools and materials, and life-cycle-oriented process design. We conclude by outlining how a closed-loop CMP ecosystem—linking materials innovation, mechanistic understanding, in situ sensing, and intelligent optimization—may support the next generation of high-efficiency, green, and manufacturable planarization technologies.

## 1. Introduction

Driven by the rapid growth of artificial intelligence (AI) and high-performance computing (HPC), demand for next-generation computing continues to expand [1,2]. The International Roadmap for Devices and Systems (IRDS) highlights the continuing requirements for higher semiconductor performance and energy efficiency [3]. At the manufacturing level, continued device scaling has driven the evolution from FinFETs toward gate-all-around nanosheet transistors [4]. Increasingly complex device architectures and heterogeneous integration are placing more stringent requirements on global planarity, local topography control, and defect suppression [5]. These developments make precise planarization increasingly important in advanced wafer fabrication. CMP has therefore remained a key enabling planarization technology in integrated circuit manufacturing, providing global surface planarization while supporting stringent requirements for surface quality and process reliability [6,7,8].

**Figure 1:**
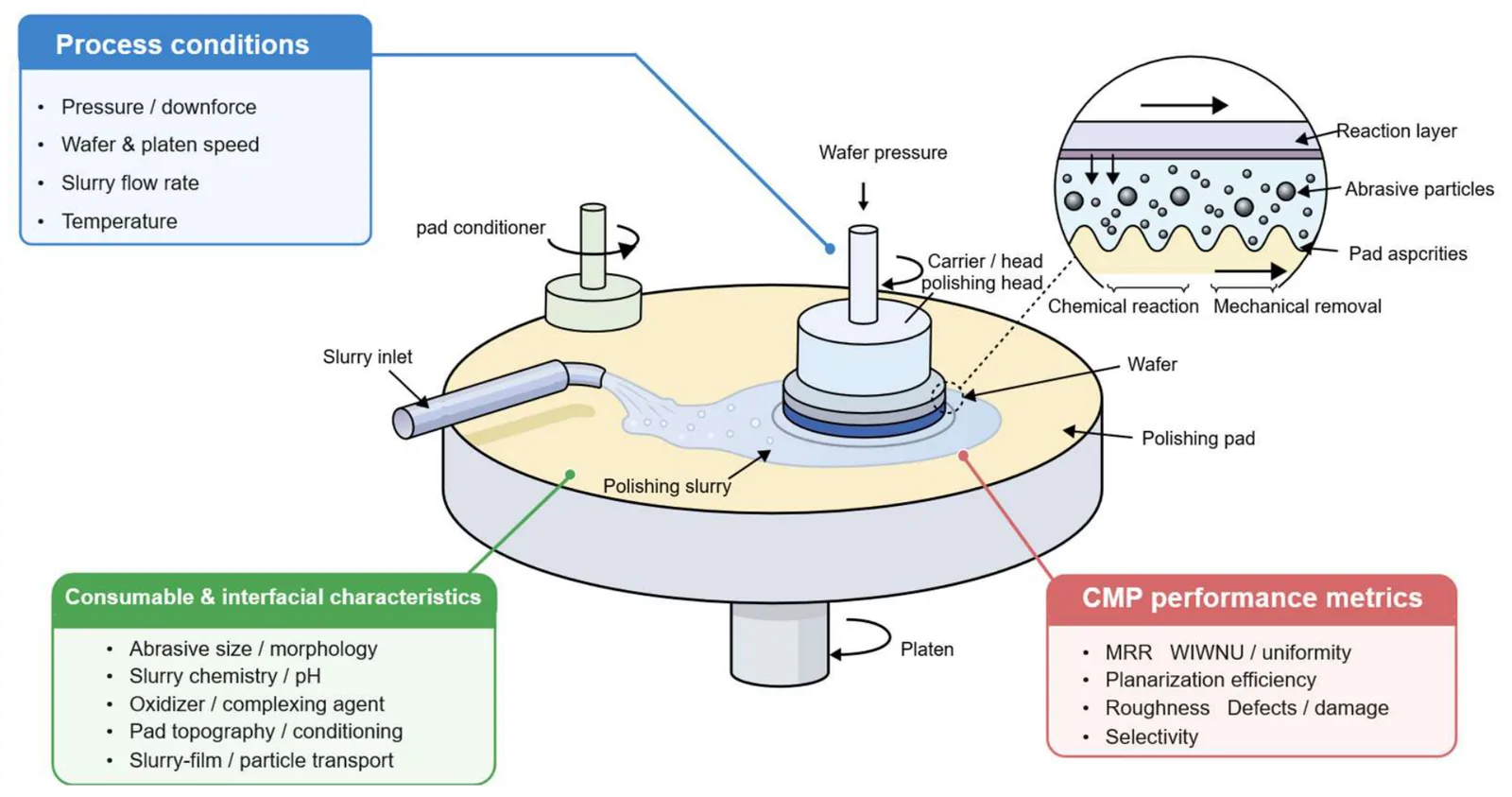
Schematic of the CMP process and key parameters governing polishing performance.

CMP is a global planarization technology that relies on the synergy between surface chemical reactions and mechanically assisted material removal [9]. A typical CMP system consists of a wafer carrier, a rotating platen covered by a polishing pad, and a slurry delivery unit. During processing, the wafer is pressed against the moving pad while slurry containing nano-abrasives and chemically active additives is continuously supplied to the interface. The process can be understood as the coupling of three highly interdependent subprocesses [8]. First, chemical modification forms a softened or chemically altered surface layer with a lower removal barrier. Second, pad asperities and abrasives mechanically shear off this modified layer, with protruding regions experiencing higher local pressure and therefore faster removal than recessed regions. Third, fluid transport continuously replenishes reactants, evacuates byproducts, and contributes to heat removal and thermal regulation at the pad–wafer interface [10,11]. CMP performance is therefore evaluated not only by material removal rate (MRR) [6,12], but also by within-wafer non-uniformity (WIWNU) [13,14], planarization efficiency [15,16], surface defects [17], and roughness evolution [18,19]. In this review, MRR is used to characterize removal efficiency, whereas surface quality refers more broadly to the overall condition of the polished surface, as reflected by defect- and roughness-related outcomes. Surface morphology describes the geometric form and spatial organization of surface features, while roughness specifically characterizes surface-height variations and is commonly quantified using parameters such as Ra, Sa, and Sq. The overall CMP process and the principal process, consumable, and performance variables are summarized in Figure 1.

The outcome of CMP is jointly determined by the chemical–mechanical characteristics of consumables and by process parameters such as pressure, relative velocity, temperature, and slurry supply conditions [20,21]. With the rapid adoption of hard and chemically inert materials, including third-generation semiconductors such as GaN [22] and SiC [23], the process window must now satisfy much higher requirements for selectivity, uniformity, and low-damage finishing. On the consumables side, researchers have developed new slurry systems by tailoring abrasive morphology [24] and porous structure [25], optimizing abrasive–slurry formulations [26] and complexing agents [27], or designing composite abrasive architectures, including CeO_2_–TiO_2_/PS–CeO_2_ systems [28], PS@CeO_2_–nanodiamond abrasives [29], and Al_2_O_3_@MIL-100 composites [30]. On the process side, external fields such as ultrasound [31], electrochemical activation [32], and photocatalysis [33] have been introduced to lower reaction barriers and facilitate removal of passive or ultra-hard surfaces [34]. Together, these developments form the present foundation of CMP materials and process innovation.

Alongside continuous process innovation, theoretical descriptions of CMP have evolved from simple empirical relationships to multiscale physical models. The Preston equation provided the earliest and most widely used phenomenological relationship between MRR, pressure, and velocity [35]. To better understand the underlying mechanisms, subsequent studies introduced models based on contact mechanics [36], CMP-specific mathematical descriptions [37], pad-texture analysis [38], fluid dynamics [39], reaction kinetics [40], and molecular dynamics (MD) simulations [41,42,43]. These approaches aim to quantify the chemical–mechanical coupling within the pad–abrasive–wafer microcontact zone, clarify the microscopic origin of material removal, and provide a predictive basis for MRR, defect generation, and planarization behavior.

Machine learning has become an important data-driven tool in intelligent manufacturing because of its ability to capture nonlinear relationships and extract useful information from high-dimensional process data [44,45]. These capabilities are particularly relevant to CMP, where strongly coupled process conditions, consumable states, equipment drift, and sensor variability complicate conventional modeling. Recent CMP studies have demonstrated machine-learning-based prediction of material removal rate using multimodal process information and pad-state descriptors [46,47], as well as virtual-metrology approaches for predicting wafer-level removal and non-uniformity from industrial process data [48]. Together, these developments indicate the growing practical role of machine learning in CMP prediction, monitoring, and process control.

Previous reviews have provided valuable perspectives on specific aspects of CMP, but a unified view connecting materials and process innovation, multiscale mechanistic understanding, data-driven intelligence, and sustainability is still limited. This review integrates these elements within a common framework and emphasizes their cross-scale connections and complementary roles in the development of intelligent CMP. Accordingly, the central question addressed in this review is how advances in materials and process innovation, multiscale mechanistic modeling, data-driven intelligence, and sustainability can be integrated to support intelligent and closed-loop CMP. In particular, it examines how mechanistic modeling, process data, virtual metrology, and intelligent control can be linked with consumable design and sustainability considerations toward a closed-loop CMP ecosystem. Based on this perspective, the review examines recent advances and remaining gaps in these areas while identifying future directions toward intelligent and sustainable CMP.

**Figure 2:**
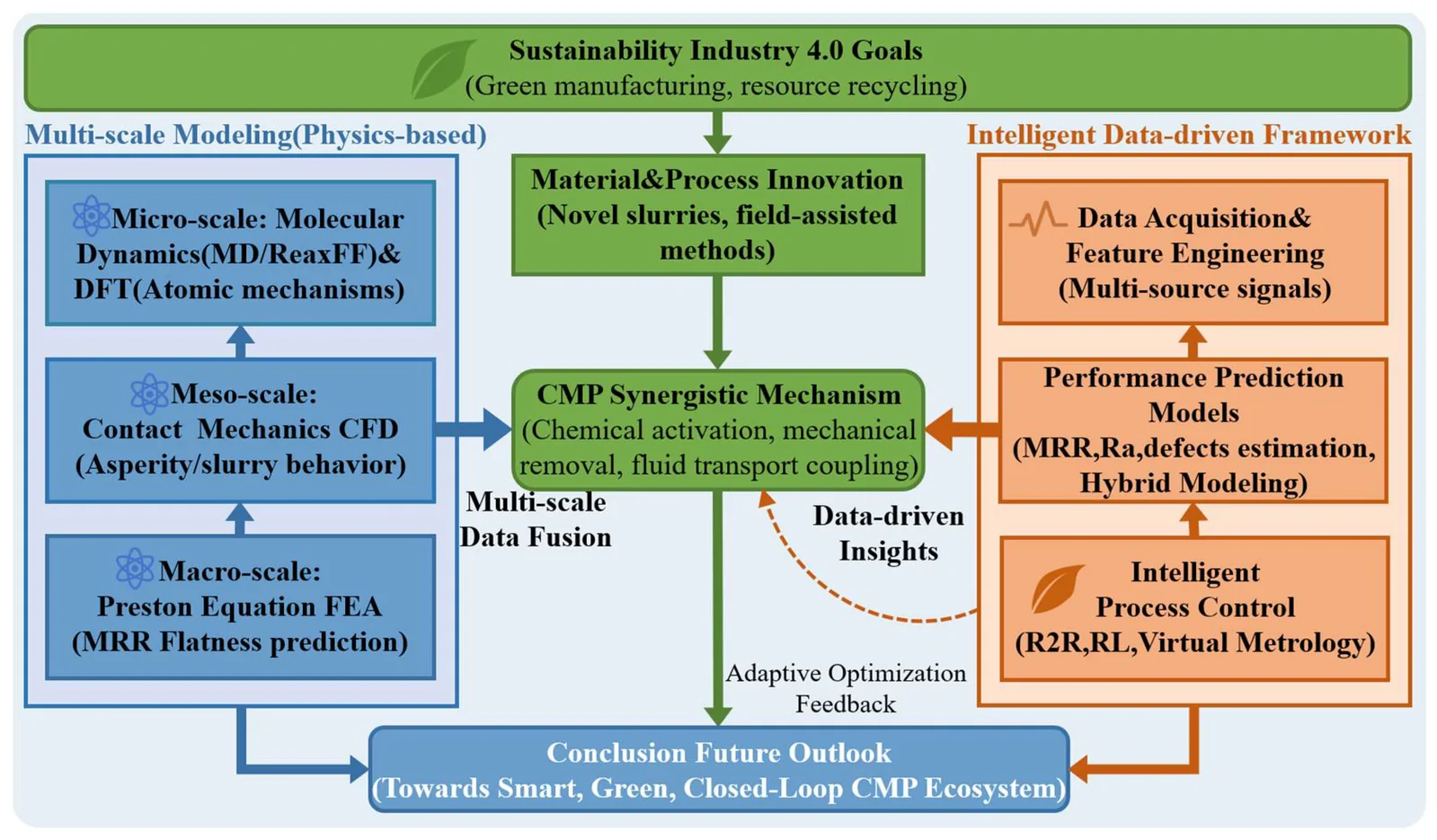
Integrated research framework for CMP in the Industry 4.0 era. The framework combines physics-based multiscale modeling—from atomistic simulation to continuum-scale analysis—with intelligent data-driven methodologies including data acquisition, performance prediction, and process control, thereby supporting materials innovation, adaptive optimization, and the development of a smart, sustainable, and closed-loop CMP ecosystem. Solid arrows indicate the principal process and information links among the framework components, whereas the dashed arrow denotes data-driven feedback or insight.

Accordingly, this review focuses on the intersection of mechanistic modeling and machine learning while also addressing advances in materials innovation and sustainability. Section 3 summarizes recent progress in slurry and process innovations, including new abrasive architectures and assisted CMP strategies. Section 4 reviews the development of multiscale modeling, from macroscale planarization models to mesoscale contact and transport descriptions and atomistic reaction simulations. Section 5 discusses the application of machine learning in CMP, particularly for performance prediction, virtual metrology, monitoring, and intelligent control. Section 6 addresses the sustainability dimension of CMP, including recycling, source reduction, and digital approaches to greener manufacturing. Section 7 concludes with major research gaps and future directions for next-generation CMP. The integrated organization of these research dimensions is summarized in Figure 2.

## 2. Review Scope and Methodology

The literature included in this review was identified through major scientific databases and literature platforms, including the Web of Science Core Collection, Scopus, IEEE Xplore, and SpringerLink. These sources provide broad coverage of peer-reviewed research relevant to CMP materials and processes, multiscale modeling, machine learning, process monitoring and control, and sustainable manufacturing.

The reviewed literature primarily focuses on studies published from 2020 to 2026, which account for approximately three quarters of the references considered in this review. Earlier publications were selectively included to establish essential theoretical and methodological foundations, including classical removal-rate, contact-mechanics, transport, and process-control models.

**Figure 3:**
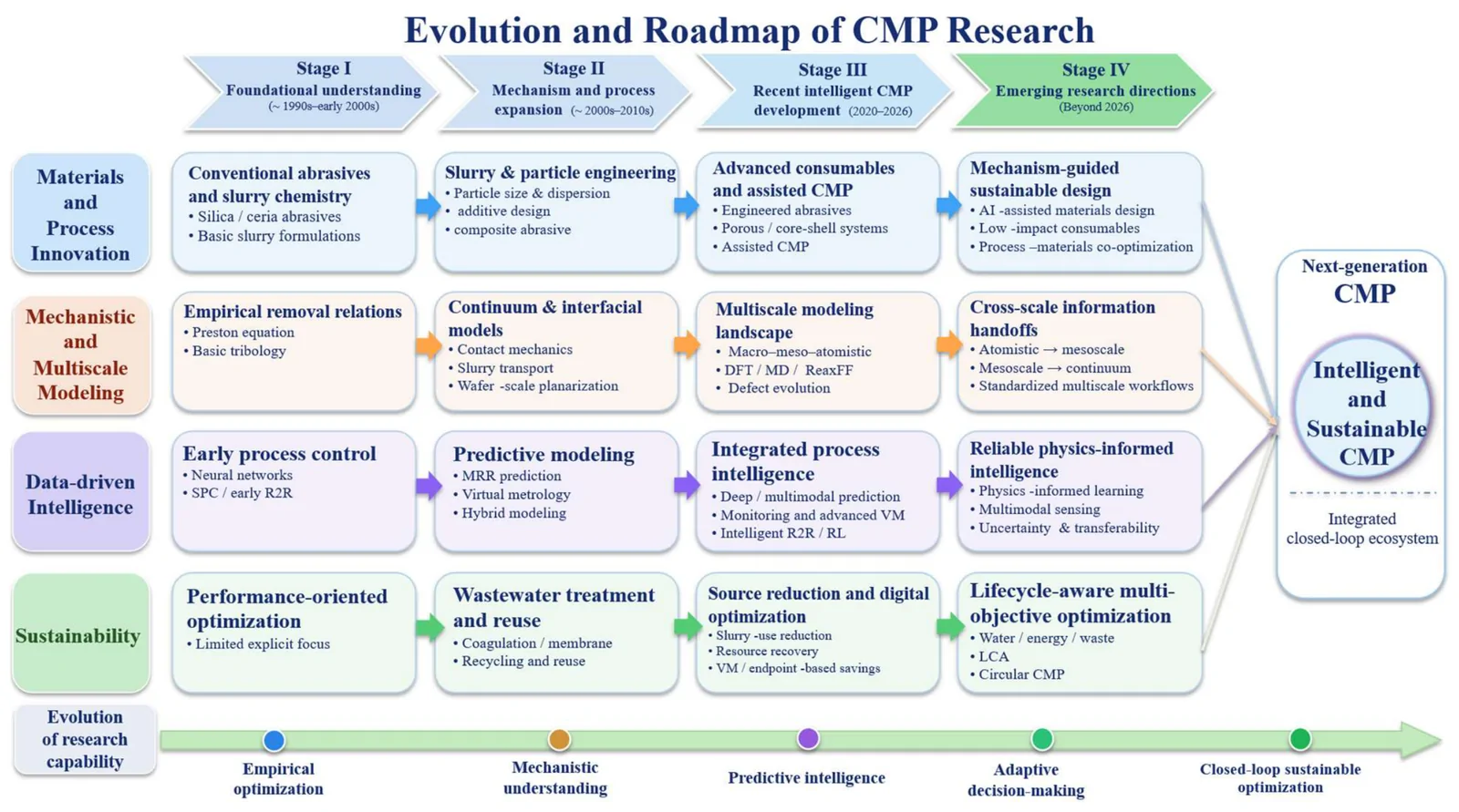
Evolution and roadmap of CMP research toward intelligent and sustainable manufacturing.

To further clarify the evolution of the field, Figure 3 summarizes the research roadmap of CMP across four interconnected dimensions: materials and process innovation, multiscale mechanistic modeling, data-driven intelligence, and sustainability. This roadmap illustrates how CMP research has progressed from empirical optimization to mechanistic understanding, predictive intelligence, adaptive decision-making, and, ultimately, intelligent and sustainable closed-loop manufacturing. It also provides the conceptual basis for the organization of the following sections.

## 3. Advances in CMP Materials and Process Technologies

In recent years, major advances in CMP have been achieved through simultaneous innovation in slurry design and process methodology. A wide range of abrasive systems have been developed to improve removal efficiency while suppressing defects, and several assisted polishing strategies have been introduced to broaden the process window, especially for ultra-hard or chemically inert materials. This section summarizes representative developments in these two directions.

### 3.1. Development of Slurry Systems

Recent abrasive optimization has focused primarily on morphology engineering and structural design. For example, in the polishing of ZrO_2_ ceramics, Xu and Lei reported that peanut-shaped and heart-shaped SiO_2_ abrasives achieved an arithmetic mean surface roughness (Ra) of 1.9 nm together with an MRR of 0.31 μm/h; the MRR was approximately 100% higher than that obtained with conventional spherical silica abrasives [24]. Their contact-wear model, supported by friction-coefficient measurements, indicated that the non-spherical particles produced multi-point and extended surface contact with the ZrO_2_ surface, increasing the effective contact area and mechanical interaction compared with conventional spherical abrasives [24]. In systems that require finer control of particle compliance and contact mode, porous [49] and ordered mesoporous abrasives [25] have attracted increasing attention. Their key advantage lies in enlarging the effective contact area, reducing local stress concentration, and enabling micro-deformation or multi-point contact under load, thereby mitigating ploughing and brittle fracture. At the same time, more complex pore structures can weaken abrasive mechanical strength, reduce batch-to-batch consistency, and destabilize slurry dispersion, making particle-size distribution control and dispersion chemistry essential for practical deployment.

**Figure 4:**
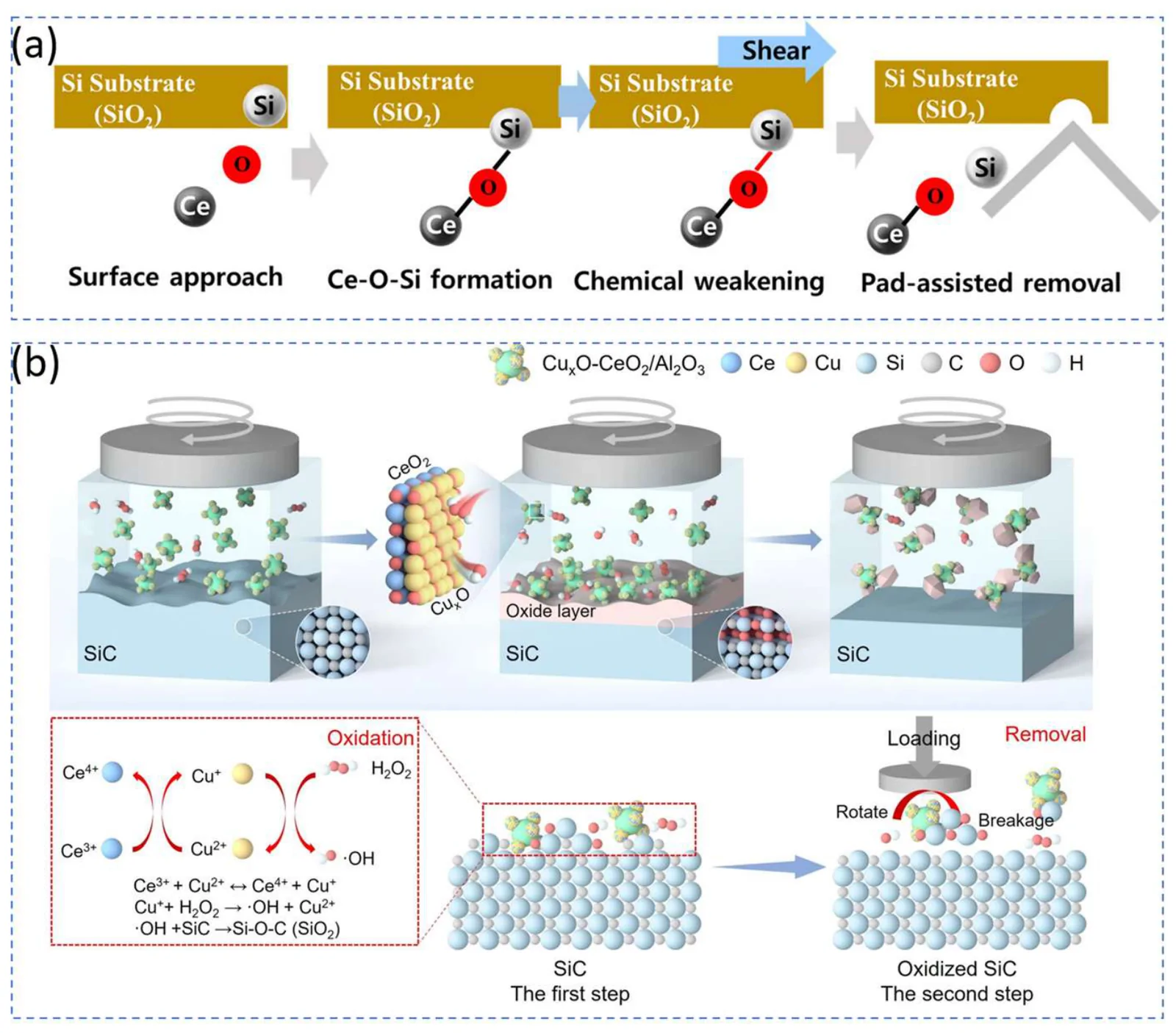
Representative interfacial and synergistic action mechanisms of advanced CMP abrasives: (**a**) Ce–O–Si interfacial bonding and chemical weakening of the SiO_2_ surface followed by pad-assisted removal, adapted from Ref. [53] under the CC BY 4.0 license, and (**b**) synergistic polishing mechanism of Cu_x_O–CeO_2_/Al_2_O_3_ composite abrasives for SiC CMP, integrating Fenton-like oxidation with mechanical abrasion, adapted from Ref. [54] under the CC BY 4.0 license.

Beyond shape control, composite and core–shell abrasives have become an important design theme. These architectures typically assign chemical reactivity to the shell while preserving structural support or compliance in the core. In sapphire CMP, Zhou et al. reported that PS@CeO_2_/DND ternary abrasives achieved an MRR of approximately 1.4–1.7 μm/h while reducing the surface roughness to Ra = 0.52 nm [29]. Similar photo-assisted SiC CMP systems have employed ceria-coated diamond core–shell abrasives [50] and TiO_2_-based heterostructured composite abrasives [51]. More recently, metal–organic framework (MOF)-based strategies have been introduced to tune surface chemistry more precisely. Representative systems include Al_2_O_3_@MIL-100 [30] and CeO_2_@MOF-derived abrasives [52]. These structures enhance interfacial reactivity through combined structural design and defect engineering. However, their practical application is still constrained by complex synthesis routes, coating non-uniformity, and the need to verify long-term dispersion stability and wear resistance under realistic polishing conditions. Representative interfacial and synergistic action mechanisms of chemically active and multifunctional abrasives are illustrated in Figure 4.

Among the most intensively studied abrasives, ceria has evolved from a physically tuned polishing medium into a chemically engineered reactive platform. Current work tunes ceria reactivity through rare-earth doping [49], composite rare-earth-oxide systems [55], CeO_2_/Y_2_O_3_ slurry design [56], porous self-assembly [49], and simplified synthesis strategies [57]. Increased surface defect density promotes oxygen migration and electron transfer, facilitates Ce^4+^/Ce^3+^ redox cycling, and enhances the formation of interfacial Ce–O–Si bonds, which in turn accelerates the formation and removal of surface reaction layers [52,56]. For example, in SiO_2_-film CMP, Zhang et al. reported that La-doped porous self-assembled CeO_2_ abrasives achieved an MRR of 338 ± 7.54 nm/min, approximately 35% higher than that of undoped porous CeO_2_, while achieving a surface roughness of Ra = 0.201 ± 0.010 nm [49]. Yet excessive surface activity may also alter selectivity or intensify local corrosion, so current formulations increasingly co-design ceria activation with inhibitors and complexing agents to balance efficiency, selectivity, and surface integrity [55].

The benefits of advanced abrasives can only be realized when they are integrated into a chemically balanced slurry formulation. To achieve atomically smooth and damage-free surfaces, recent formulations typically follow an oxidation–complexation–removal sequence [12]: an oxidizer generates a chemically modified surface layer, a complexing agent promotes dissolution and transports dissolved species away, and mechanical action strips the altered layer while minimizing redeposition and pitting. In metals and interconnect structures, these subprocesses are strongly coupled because pH, ionic strength, and interfacial charge simultaneously influence corrosion, adsorption, and particle–surface interaction. For example, in Co/Ti interconnect CMP, potassium citrate promotes dissolution of the Ti barrier while also modifying the interfacial electrostatic environment, thereby facilitating both chemical dissolution and mechanical removal [27]. Similar strategies have been demonstrated for Cu [58], Ni alloys [59], and aluminum alloys [60], where balanced oxidizing and complexing chemistry can improve removal while suppressing defects [26,60], and for zinc germanium phosphide, where relatively mild green chemistries have enabled atomic-scale finishing with reduced deep damage [61]. These results suggest that future slurry design should optimize oxidation strength, complexation capacity, and suspension stability together rather than maximizing MRR alone.

To further suppress defects, many recent studies have introduced dispersing or lubricating additives such as h-BN [62], graphene oxide [32], MoS_2_ [56], and polymeric dispersants [63]. These components help stabilize particle dispersion and reduce interfacial friction, thereby lowering the probability of scratches while preserving an acceptable removal rate. However, their dosage must be carefully controlled: excessive lubricating or polymeric coverage can weaken abrasive-surface contact and suppress interfacial reactions, eventually reducing MRR. Mixed-abrasive systems provide another route to balancing efficiency and surface quality. By combining ceria with diamond, silica, or other particles, researchers have achieved synergistic tradeoffs and have begun to develop more systematic guidance for formulation selection across material systems [64].

### 3.2. Emerging Assisted CMP Technologies

**Figure 5:**
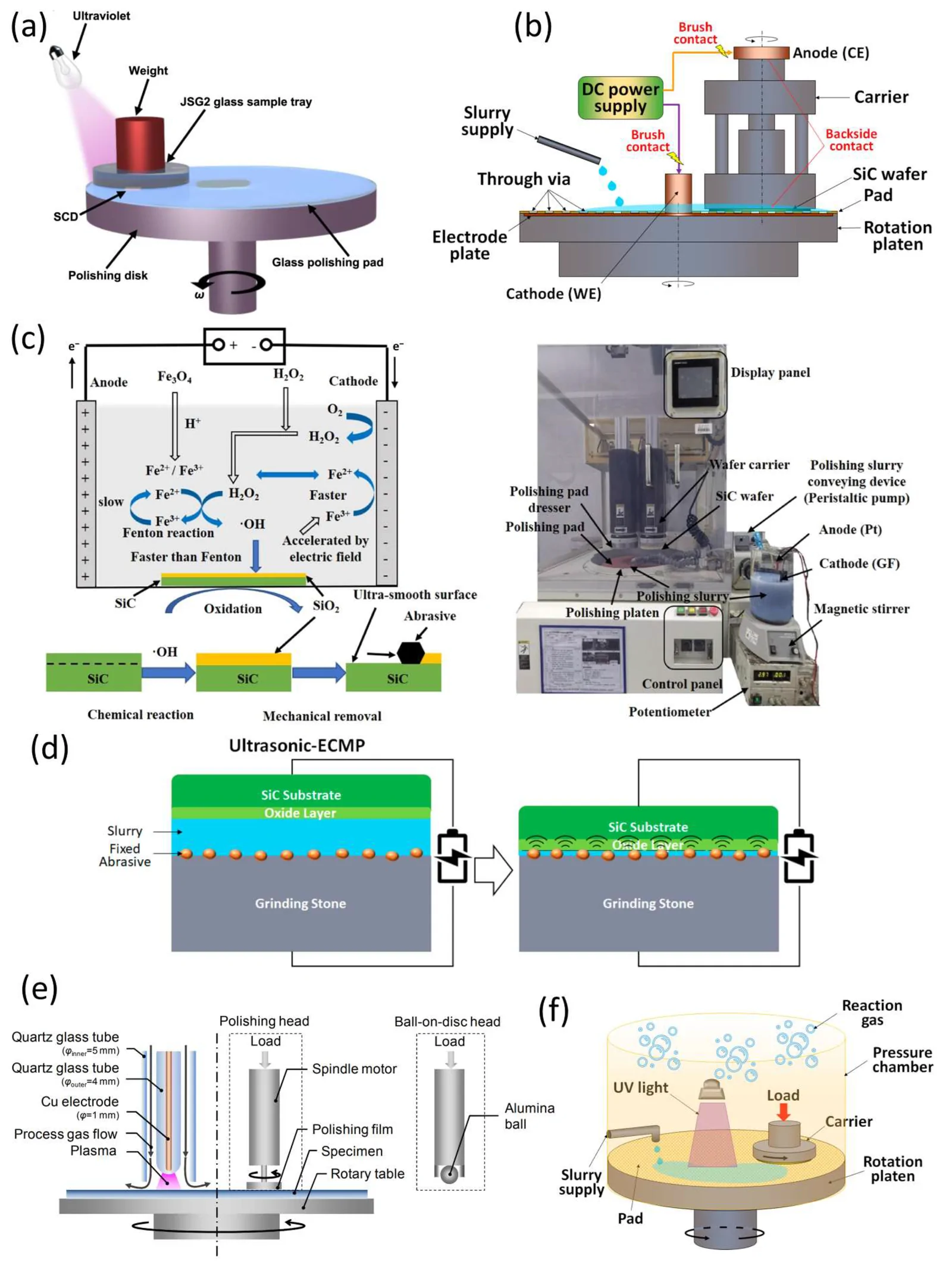
Schematic diagrams of representative assisted CMP technologies: (**a**) PCMP [65]; (**b**) ECMP [69]; (**c**) EF-CMP [70]; (**d**) ultrasonic-assisted electrochemical mechanical polishing [69]; (**e**) plasma-assisted CMP [71]; and (**f**) gas-assisted CMP [69]. Panels (**a**–**f**) were adapted from Refs. [65,69,70,71] under the CC BY 4.0 license.

For ultra-hard and chemically inert materials such as SiC, diamond, and sapphire, conventional CMP often suffers from low reaction efficiency and poor control of subsurface damage. To overcome these limitations, various externally assisted CMP technologies have emerged. By introducing UV-assisted activation for SiC [23], photoelectrochemical assistance [28], photocatalytic routes for diamond [65] and YAG [66], electro-Fenton assistance [20] or electrochemical polishing with graphene oxide [32], ultrasonic vibration [67], plasma [34], or gas-atmosphere regulation [68], often together with radical-generating chemistries [30], these methods accelerate the formation and renewal of surface reaction layers so that thinner and more uniform modified layers can be removed more steadily by abrasives. Representative process concepts include photocatalytic CMP (PCMP), electrochemical mechanical polishing (ECMP), photoelectrochemical mechanical polishing (PECMP), ultrasonic-assisted electrochemical polishing, plasma-assisted CMP, and gas-assisted CMP. Representative schematics of these assisted-CMP technologies are shown in Figure 5.

Recent studies on 4H-SiC have reported substantial improvements in polishing efficiency and surface quality through photo- and electro-assisted processes. Gao et al. demonstrated PECMP in which external bias and illumination jointly promoted formation of a removable oxide layer; under the optimized conditions, the process achieved an MRR of 1.109 μm/h with Ra = 0.738 nm, while a further verification test produced a scratch-free surface with Ra = 0.113 nm [28]. Wang et al. activated persulfate using TiO_2_ and ultraviolet irradiation, quantitatively correlating UV intensity and TiO_2_ loading with radical generation, MRR, and roughness, and showing that moderate catalyst content can balance efficiency and surface quality [23]. Zhu et al. improved SiC planarization through photocatalytic-assisted CMP using ceria-coated diamond core–shell abrasives [50], whereas Chen and Lei employed TiO_2_-based heterostructured composite abrasives for photo-assisted CMP of 4H-SiC [51]. On the electrochemical side, Deng et al. employed an electro-Fenton system to generate hydroxyl radicals in situ near the anode, obtaining high MRR and low roughness under relatively low mechanical load [20], whereas Wang et al. combined ECMP with graphene oxide and diamond abrasives to improve lubrication and dispersion while maintaining near-atomic smoothness [32]. Related work on ultrasonic-assisted core–shell abrasive CMP [67] and ultrasonic vibration-assisted ECMP [72] for SiC likewise showed that the added field can increase efficiency, although excessive amplitude may thicken residual oxide layers or aggravate surface texturing.

For diamond, YAG, sapphire, and related hard crystals, assisted processes mainly enhance oxidation and interfacial wetting so that removable surface layers can be formed under milder mechanical conditions. Guo et al. used a UV-PMS photocatalytic system to increase the oxidation rate and reduce damage during YAG polishing [66]. Xu et al. showed that increasing the dissolved-oxygen concentration to 40 ppm reduced the sapphire surface roughness by 57.3% to Ra = 0.124 nm while increasing the MRR by 23.02% relative to conventional CMP [68]. Lyu et al. reported a plasma-assisted route for Lu_2_O_3_ that achieved a plasma modification rate of 136 nm/h and an atomically smooth, subsurface-damage-free surface with Sa = 0.14 nm, while reducing the overall processing time to approximately half that of low-pressure slurry polishing [73]. In a different but related strategy, Wu et al. reported friction-induced chemical reaction polishing of single-crystal 4H-SiC using pure iron, where frictional heating and tribochemistry generated a removable reacted layer and enabled a removal rate of 375 nm/min together with an Ra of 2.2 nm [74]. Across these material systems, the main value of assisted CMP lies not simply in increasing MRR, but in broadening the process window, reducing damage, and stabilizing surface quality.

Despite their demonstrated advantages on blanket wafers, most assisted CMP studies remain far from production deployment. The majority of current experiments are performed on unpatterned substrates, whereas real device topographies involve trenches, steps, and multilayer stacks in which local field distribution and reaction heterogeneity may induce dishing, line-width variation, and residual-thickness errors. Assisted processes also introduce new concerns regarding equipment durability, consumable compatibility, and environmental or safety risk—for example, electrode stability, pad degradation by strong oxidants or plasma, and long-term effects on plumbing and waste treatment. Future work should therefore move beyond proof-of-concept demonstrations on flat wafers and systematically evaluate long-term stability, defect statistics, and process windows on patterned structures relevant to manufacturing.

To facilitate quantitative comparison across representative materials and assisted CMP strategies, Table 1 summarizes the reported processing conditions and key performance metrics, including MRR, surface roughness, and defect- or selectivity-related indicators. Because the substrate materials, polishing conditions, and measurement methods differ among studies, the reported values are intended for structured comparison.

## 4. Multiscale Modeling of CMP

In this review, multiscale modeling refers to the description of CMP phenomena across distinct but interacting characteristic length scales. Three main levels are considered. Atomistic interfacial reactions occur primarily at the ångström-to-nanometer scale. Mesoscale abrasive–pad–slurry interactions extend approximately from tens of nanometers to tens of micrometers, while feature-, chip-, and wafer-scale planarization extends from local surface features to wafer dimensions (~10^−1^ m). These ranges are approximate and may overlap because the relevant scale is determined mainly by the dominant physical process rather than by a rigid geometric boundary [75,76]. At the atomistic level, first-principles calculations and molecular dynamics resolve adsorption, reaction pathways, bond formation and rupture, and atomic removal [77]. At the mesoscale, contact mechanics, statistical asperity models, lubrication, and multiphase transport describe real contact area, local pressure, slurry-film behavior, particle transport, and local removal [8]. At the feature-to-wafer scale, continuum pressure/velocity-field and Preston-type models predict profile evolution, dishing, thickness variation, and within-wafer uniformity [76]. All three scales are significant for different modeling objectives, while the mesoscale often serves as the practical bridge between atomistic interfacial mechanisms and wafer-scale removal behavior.

Multiscale coverage should, however, be distinguished from multiscale integration. In this review, integration refers to cases in which information or physical relations obtained at one scale are explicitly transferred to or coupled with a model at another scale, consistent with the general information-passing concept of multiscale modeling [78]. Atomistic calculations can provide reaction energetics, bonding characteristics, or parameters for reactive force fields and effective reaction descriptions. Mesoscale models then translate interfacial behavior into quantities such as real contact area, local pressure, film thickness, particle flux, and local removal functions. These quantities can enter feature- or wafer-scale models and may also provide physics-based features or constraints for data-driven prediction and control. Integrated examples already exist in CMP. Seok et al. linked abrasive-particle, pad-asperity, and wafer scales through contact relations and statistical pressure/removal calculations [79]. More recently, Zhao et al. integrated fiber-based modeling, Eulerian–Lagrangian analysis, and ReaxFF-MD in a millimeter-to-nanometer cross-scale framework [80]. Nevertheless, most CMP studies still operate primarily within individual scales, and standardized information transfer among atomistic, mesoscale, and wafer-scale models remains limited. Accordingly, this section reviews these scales separately while emphasizing their modeling roles and the opportunities for cross-scale integration.

### 4.1. Macroscale CMP Models

At the wafer or chip scale, the main modeling targets are thickness distribution, planarity evolution, and process uniformity. Macroscale models do not usually attempt to resolve single-particle reaction events; instead, they start from inputs such as nominal load, relative velocity distribution, and carrier-pad contact state, and then predict the accumulated material removal over time. The Preston relation remains the most widely used starting point for such analyses and is commonly written as MRR = k_p_PV, where P is nominal pressure, V is relative velocity, and k_p_ is the Preston coefficient that lumps together the effects of pad, slurry, and interfacial conditions [35]. In real CMP, however, k_p_ is not constant and evolves with consumables and interface states [8,19]. Recent macroscale modeling therefore retains the Preston form while linking its coefficient to velocity fields, pad wear, surface microstructure, or assisted-field conditions in order to improve predictions of planarization, profile evolution, and within-wafer uniformity.

For wafer-scale flatness prediction, Zhao et al. used Ansys-Fluent to compute the surface pressure distribution and relative velocity, and then combined these results with an experimentally determined Preston coefficient to predict total thickness variation (TTV) [81]. To capture finer-scale topography, Xu et al. developed a two-scale pressure framework for tungsten CMP in which chip-scale pressure describes long-range profile change while feature-scale pressure accounts for pattern effects and is coupled with steady-state oxidation kinetics to predict local dishing under different slurry conditions [82]. These studies show that macroscale modeling is no longer limited to average removal-rate estimation; it increasingly aims to resolve local planarization differences on the basis of computable pressure and velocity fields. Meanwhile, process evolution introduces additional time dependence. Jeong et al. described pad-state evolution using a wear-dependent probability density function of asperity height and used it to explain removal-rate drift over time [83]. For engineered pads and hybrid processes, such effects become even more pronounced. Yun et al. discussed how microstructured pads modify the effective Preston coefficient through geometry and operating conditions [84]. In ultrasonic-assisted sapphire CMP, Zhou and co-workers further proposed a removal model that combines two-body abrasion and particle-impact contributions, validated by in situ characterization, CFD, and polishing experiments, to explain how amplitude and rotation speed jointly affect MRR and surface quality. Overall, the strength of macroscale modeling lies in turning tool-level and process-level inputs into computable predictions of profile evolution and planarity control, although its reliability still depends strongly on how local removal laws are formulated and calibrated.

### 4.2. Mesoscale CMP Models

Mesoscale modeling focuses on statistically meaningful variables in the interfacial contact zone. It translates load, velocity, pad morphology, and abrasive features into intermediate quantities such as real contact area, load partitioning, slurry-film behavior, and local removal functions, which can then be linked to measurable outputs such as MRR, uniformity, roughness, or power spectral density. In this sense, mesoscale models serve as the bridge between wafer-scale descriptions and atomistic mechanisms. The following discussion is organized around interfacial contact mechanics, slurry flow and multiphase transport, and surface-topography evolution, all of which are mutually coupled through film thickness, particle flux, and local-removal boundary conditions.

#### 4.2.1. Interfacial Contact Mechanics

Mesoscale contact analysis in CMP typically begins with classical contact mechanics [36]. At the single-particle level, Hertzian contact theory relates local contact radius to contact area and mean pressure [85]. The Greenwood–Williamson framework extends this logic to rough surfaces by statistically relating real contact area to macroscopic load and rough-surface topography [86]. Within this perspective, the Preston equation can be interpreted as a macroscopic projection of mesoscale contact statistics and interfacial reaction rates [6,35].

Based on these foundations, recent contact models have developed along three main lines. Abrasive-dominated models treat the number of active particles and their load-sharing states as the key variables. Chen et al. systematically analyzed embedded and non-embedded abrasive contact states and revealed why very high local stress can invalidate the linear Preston relation [87]. Qin et al. further derived an efficient MRR expression by incorporating particle elastoplastic deformation, load distribution, and the number of effective contacting particles, thereby preserving physical interpretability while maintaining a compact form [17]. These models are intuitive and useful for analyzing statistical particle contributions, but their validity depends on simplified assumptions about particle engagement, embedding, and contact effectiveness.

Pad-dominated models, by contrast, emphasize the leading role of pad surface topography. Wang et al. developed a pad-asperity-scale physical model that quantifies chemical–mechanical synergy through physically meaningful parameters and statistically calibrates contact-spot distributions using measured pad surface topography [88]. In subsequent work, they advanced from simple contact-area ratios to effective contact-spot statistics such as spot size, spacing, and distribution, and coupled these with chemically and mechanically weighted capability parameters to interpret nonlinear MRR responses under different conditions [89]. Yun et al. later extended this line of thinking from randomly rough pads to periodic microstructured surfaces and showed that the Preston coefficient becomes a predictable function of pad geometry and operating conditions rather than a purely empirical constant [84]. The strength of pad-dominated modeling is that measured pad surface topography can be used directly as an input, making it easier to rationalize nonlinear removal behavior and within-wafer uniformity. Its limitation is that key parameters usually require system-specific calibration, and contact statistics alone cannot fully explain behavior when slurry renewal and species transport play dominant roles.

A third category involves multi-coupled contact models that explicitly integrate chemistry, abrasives, and external-field effects. Xia et al. decomposed material removal into chemically mediated dissolution and mechanically assisted stripping, allowing the model to transition smoothly across chemistry-dominated and mechanics-dominated regimes [40]. Song et al. [90] and Zheng et al. [91] further introduced atomistic insight and chemical-bonding concepts into MRR modeling for diamond and tantalum CMP, respectively, thereby clarifying how bond formation and rupture contribute to the overall chemical–mechanical synergy. Such models broaden applicability but also increase parameter complexity, making calibration more difficult and highlighting the need for validation across wider operating windows.

Taken together, mesoscale contact models have evolved from simple elastoplastic contact descriptions into multi-variable systems capable of integrating abrasive statistics, pad morphology, and surface chemistry. They consistently suggest that coordinated optimization of effective contact sites, load partitioning, and interfacial reactivity is central to improving both MRR and surface quality. Two challenges nevertheless remain. First, model calibration still relies heavily on discrete experiments, which limits convenience and generality. Second, contact models are often only weakly coupled to slurry flow and mass transport, making it difficult to close the dynamic balance of reactant supply, byproduct removal, and interfacial renewal during polishing.

#### 4.2.2. Slurry Flow and Multiphase Transport

Slurry flow governs the distribution and renewal of abrasives and chemical species within the thin fluid film between the pad and the workpiece, thereby influencing reaction continuity, debris evacuation, and ultimately uniformity and process stability [13,92,93,94,95]. Modeling of this process relies mainly on computational fluid dynamics (CFD), sometimes combined with additional physical fields to represent assisted CMP processes.

For direct simulation, researchers typically work within lubrication theory or fully three-dimensional Navier–Stokes formulations [96]. Lin and Liao used three-dimensional CFD to quantify how process parameters affect slurry residence time, providing a basis for identifying insufficient slurry replenishment [92]. To capture particle-transport details, Zhou et al. applied a dense discrete phase model to sapphire CMP and introduced the particle erosion ratio (PER) to quantify the contribution of individual abrasives. They found that increasing particle concentration can intensify shielding and particle–particle interference, so that some particles no longer interact effectively with the reacted surface layer, causing PER to decrease even when nominal concentration rises [93]. Sadri Mofakham et al. compared different pad microstructures through CFD and experiments and showed that a square-with-circle pattern with a larger designed inverse contact length did not produce the greatest pressure drop, but did generate stronger particle enrichment at the upstream edge together with more uniform velocity and moderate residence time, which translated into improved MRR and within-wafer uniformity [39].

To extend CFD to emerging hybrid processes, recent work has increasingly coupled flow fields with other physical fields. Zhou et al. developed a gas–liquid two-phase CFD model for ultrasonic vibration-assisted CMP and compared velocity, pressure, and air-volume fraction distributions under different amplitudes. Their results showed that cavitation bubble collapse can generate localized high temperature and pressure, thereby affecting both surface reactions and local removal, while excessively large amplitude increases the air fraction and reduces the liquid fraction, which can eventually weaken the polishing effect [97]. Wu et al. further coupled acoustic and flow fields to analyze ultrasound-induced microstreaming and cavitation, providing a physical explanation for why high removal capability can be maintained even at relatively low nominal loads [94]. These studies demonstrate that slurry transport is not merely a background phenomenon; it is a dynamic mediator of particle effectiveness, chemical supply, and assisted-field coupling.

#### 4.2.3. Surface Topography Evolution

Compared with local contact mechanics and interfacial flow analysis, surface-topography evolution models connect more directly to experimentally measurable outputs such as roughness and power spectral density (PSD). They therefore provide a critical bridge between mechanism and metrology. In recent years, the emphasis has shifted from empirical fitting toward physically interpretable numerical frameworks whose outputs can be compared directly with surface-characterization data. Xia et al. developed a microscale removal model that simultaneously predicts roughness and PSD evolution within a unified framework [98]. Yang et al. further introduced a reaction-layer-based surface renewal model in which partial and complete removal of the reacted layer are treated separately, enabling a mechanistic explanation of how roughness and PSD evolve differently under different process conditions [99].

As a complement to workpiece-focused models, Wang et al. incorporated pad asperities and pore features into surface-profile prediction for fixed-point polishing, using measured pad morphology to determine contact-related statistics and the number of effectively participating particles, and thereby to construct the spatial distribution of local removal [100]. Such approaches establish a direct relationship between measurable pad morphology and local removal functions, allowing topography evolution to reflect changes in interfacial contact rather than merely updating the workpiece surface in isolation. Both modeling and experimental characterization indicate that increasing abrasive size or slurry pH often accelerates convergence of roughness and PSD, but can also raise the steady-state PSD level [13,99]. The relationship between surface topography and functional response can also depend strongly on the spatial scale at which the surface is characterized. Scale-sensitive analyses of microtextured surfaces have shown that such correlations may reach a maximum within a characteristic observation range. For example, Peta reported that the correlation between surface fractal complexity and wettability was strongest at an area scale of 1024 μm^2^ and a length scale of 32 μm, whereas both finer and coarser observation scales produced weaker correlations [101]. Subsequent neural-network modeling further demonstrated that surface descriptors selected according to their strong functional correlations can support accurate prediction of surface response [102].

Scale-resolved quantities, such as wavelength-dependent PSD features, asperity statistics, and filtered topographic descriptors, could therefore be extracted over a series of spatial scales and compared with the target response. In practice, measured pad or wafer topography could be analyzed using different filtering lengths, spatial-frequency bands, or observation windows, after which PSD-derived features, asperity density, asperity radius, or roughness parameters are calculated at each scale. The characteristic scale range could then be identified as the interval in which these descriptors show the strongest and most stable correlation with the target quantity, such as MRR, roughness, dishing, or within-wafer non-uniformity. The selected scale-dependent descriptors could subsequently be used as inputs to machine-learning models and further evaluated through predictive performance on unseen data. Surface descriptors have already been used as inputs to neural networks for predicting contact-mechanical responses, illustrating how physically meaningful topographic information can be incorporated into data-driven models [103]. Identifying the characteristic observation scale in this way may improve prediction accuracy by retaining the surface information most relevant to the target while reducing irrelevant fine-scale variation or excessive coarse-scale smoothing. It may also improve interpretability by linking the selected features to identifiable contact, removal, or planarization mechanisms.

Overall, topography-evolution modeling is becoming more quantitative and characterization-oriented, although its transferability still depends on how local removal laws are defined and whether contact and transport models can provide sufficiently closed boundary conditions.

### 4.3. Microscale and Atomistic CMP Models

Macroscopic outcomes such as removal rate, selectivity, and defect formation are highly sensitive to the chemical environment because they are controlled by a small number of atomistic interfacial events: whether abrasive surfaces can trigger oxidation and bridge-bond formation, whether passivation films remain stable, and whether complexing agents effectively dissolve reaction products. Atomistic modeling is therefore essential for identifying reaction pathways, active sites, and bond-breaking mechanisms that cannot be resolved experimentally in situ.

#### 4.3.1. First-Principles Calculations and DFT

First-principles calculations provide the most direct route for constraining such interfacial chemistry. Chang et al. compared the deprotonation state and adsorption structures of benzotriazole and 1,2,4-triazole on Cu/Cu_2_O–OH surfaces under different pH conditions and showed that benzotriazole can form a stable inhibiting adsorption layer already in acidic media, whereas 1,2,4-triazole becomes strongly inhibitory only in alkaline conditions; at pH 10, the two inhibitors exhibit comparable effectiveness [104]. Dang et al. calculated the reaction barriers of ethylenediamine on Si, SiO_2_, and Si_3_N_4_ surfaces in high-selectivity polysilicon CMP and found more favorable bonding on Si, thereby explaining selective removal from the standpoint of electronic structure [105]. Luo et al. combined adsorption-energy and charge-distribution analysis for polyamines on Cu/Cu_2_O/CuO surfaces with electrochemistry and diffusion simulations, and linked the number of coordination sites and adsorption strength to impedance behavior and removal rate [106]. These examples show that DFT is highly valuable for mechanism discovery and relative-performance comparison, although its predictive power is limited by the complexity of the interfacial environment and the finite size of the computational model.

**Figure 6:**
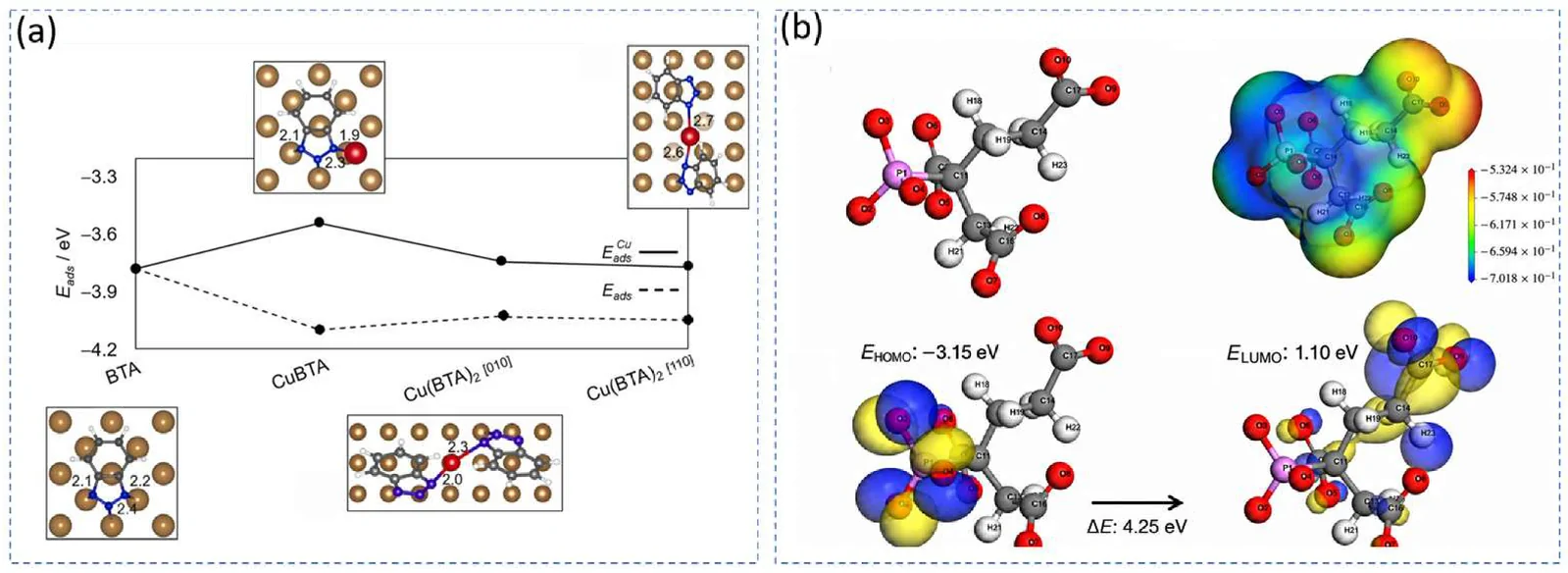
Representative DFT analyses relevant to CMP interfacial chemistry: (**a**) adsorption configurations and adsorption energetics of benzotriazolate species on Cu(100), adapted from Ref. [108] under the CC BY 4.0 license, and (**b**) electrostatic-potential and frontier-molecular-orbital analyses of PBTCA for identifying reactive coordination sites in Cu CMP, adapted from Ref. [109] under the CC BY 4.0 license.

When the focus shifts to abrasive-mediated reaction pathways, DFT is particularly effective for identifying chemically active sites. Wang et al. showed that the –COOH and –NH_2_ groups in aminobutyric acid and sodium alginate are the main functional sites participating in electron transfer and coordination with CeO_2_ surfaces and the Si–O network, in good agreement with experimental observations of high removal rate and sub-nanometer roughness [63]. Hu et al. summarized the two major roles of DFT outputs in multiscale CMP modeling: one is to parameterize reactive force fields such as ReaxFF, and the other is to provide reaction-related boundary conditions for continuum-scale descriptions [107]. In this sense, first-principles calculations are not only explanatory tools, but also an important gateway through which atomistic chemistry enters larger-scale predictive frameworks. Representative DFT analyses of adsorption energetics and reactive coordination sites relevant to CMP interfacial chemistry are summarized in Figure 6.

#### 4.3.2. Molecular Dynamics and Reactive MD

**Figure 7:**
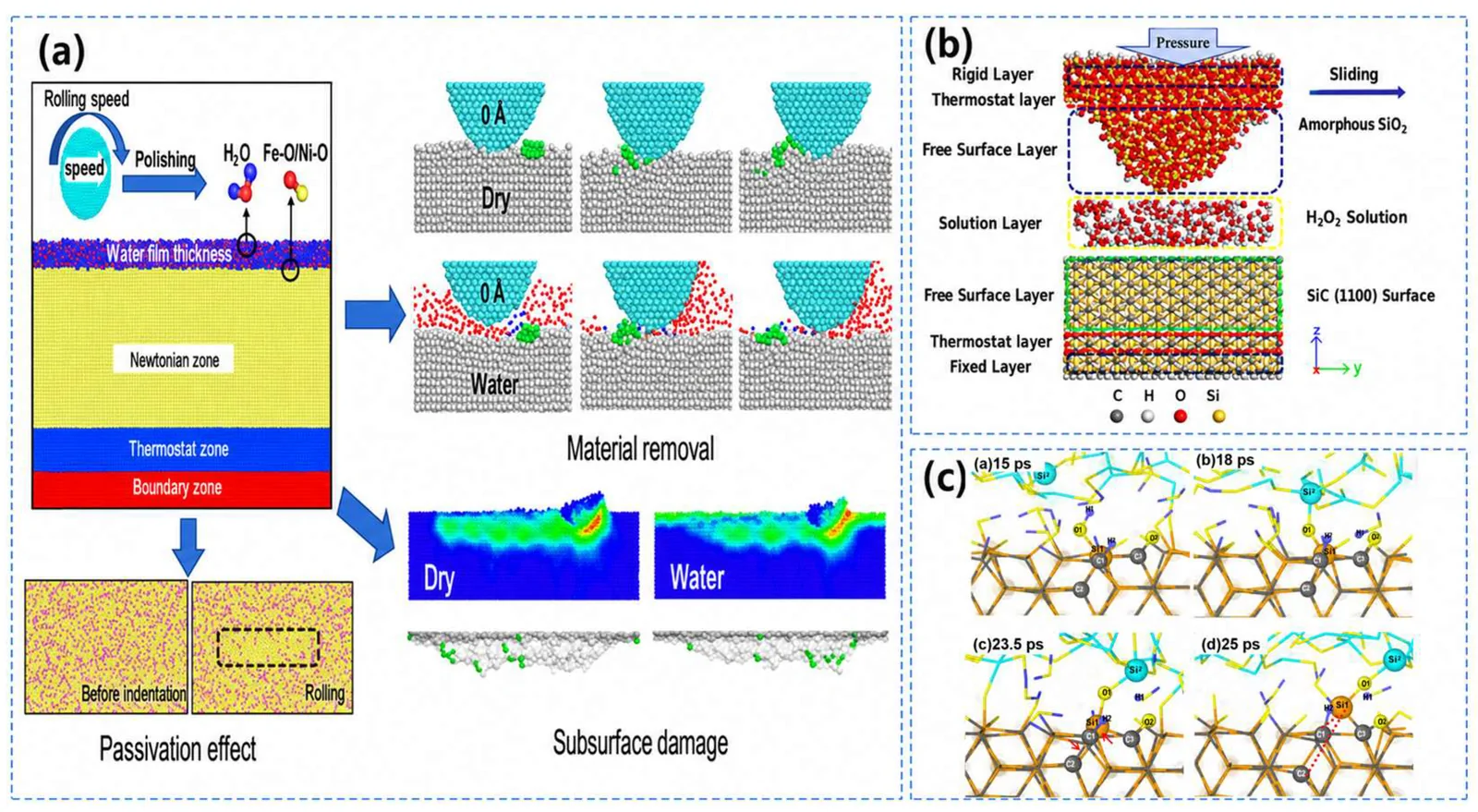
Representative atomistic MD and reactive-MD frameworks for CMP: (**a**) classical MD framework and representative water-film effects during CMP, adapted from Ref. [111] under the CC BY 4.0 license; (**b**) ReaxFF-MD model of SiC CMP in aqueous H_2_O_2_; and (**c**) atomistic Si-removal process mediated by interfacial Si–O–Si bridge bonds, with (**b**,**c**) adapted from Ref. [112] under the CC BY 4.0 license.

At the atomistic-dynamics level, conventional MD and reactive MD play complementary roles. Classical MD is mainly used to reveal how contact stress distribution, abrasive kinematics, and lubrication conditions alter damage risk and the boundaries of mechanical removal. For Si CMP, Chen et al. established a three-body pad–abrasive–workpiece model and introduced abrasive rotation, showing that moderate self-rotation can reduce local stress concentration and alleviate scratching while maintaining effective removal [43]. For 4H-SiC, Wang et al. combined experimentally measured the mechanical properties of the C-face and Si-face with MD simulations and showed that the C-face more readily releases stress through dense dislocation activity, thereby explaining the experimentally observed differences in grinding force and surface quality [110]. Do and Fang introduced surface texture and ultra-thin water-film lubrication into β-SiC polishing models and demonstrated that suitable combinations of texture parameters and film thickness can reduce residual stress and roughness without sacrificing removal [42]. Classical MD thus provides a clear picture of atomistic contact mechanics, but it cannot adequately describe bond formation, bond rupture, oxidation, or complexation, which motivates the use of reactive simulations. Representative atomistic MD and reactive-MD frameworks for CMP are shown in Figure 7.

Reactive force field molecular dynamics (ReaxFF-MD) overcomes these limitations by introducing bond-order-dependent potentials so that bond formation and rupture can be observed directly during the simulation trajectory. Recent ReaxFF studies have clarified essential mechanisms in several material systems. In glass and fused-silica polishing, Liu et al. combined ReaxFF-MD and experiments to show that mesoporous shell/core SiO_2_ abrasives can improve removal by redistributing contact stress and modifying the thickness of the reactive layer [113]. Li et al. explicitly tracked the formation and rupture of Ce–O–Si bridge bonds in a CeO_2_–H_2_O–fused-silica interface and related the bridge-bond population and the number of displaced surface atoms above a threshold to atomistic removal efficiency under different loads and sliding speeds [114]. Wang et al. further revealed oxidation and weakened-layer formation in a CeO_2_–organic-additive–quartz system, consistent with experimentally observed high removal rate and roughness near 0.1 nm [63].

For wide-bandgap semiconductors and ultra-hard materials, the main value of reactive MD lies in explaining how chemical activation can overcome intrinsic hardness. Gu et al. [41] and Zhou et al. [115]’s ReaxFF simulations of SiC CMP have shown that H_2_O_2_ promotes the formation of Si–OH and Si–O–Si structures on the surface, which reduces both friction coefficient and subsurface damage during subsequent removal. Similar simulations for fixed-abrasive polishing have suggested that alkaline salts such as Na_2_CO_3_ can accelerate oxidation and etching, thereby increasing the effective removal rate [116]. External fields can amplify these effects. In ECMP [117] and PECMP [118], electric fields or illumination increase the generation rate of hydroxyl and oxide species and deepen oxygen penetration, leading to thicker but easier-to-remove oxide layers [117,118]. At the atomistic scale, surface roughness and real contact conditions have been linked to interfacial friction in diamond CMP [119]. ReaxFF-MD has also been combined with a CPO-GMM-LSSVM model to predict atomic-scale surface integrity in polycrystalline diamond [120], while ReaxFF-MD together with experiments has been used to clarify tribochemical atom-removal mechanisms in sapphire CMP [121]. In copper CMP, ReaxFF-MD has been used to quantify single-atomic-layer removal mechanisms [122] and to characterize the distinctive atomic slipping behavior associated with atomic-layer removal [123].

Despite their diversity in materials and methodology, these atomistic studies converge on a common logic: the interface is first chemically activated to form bridge bonds or weakened layers that can transmit shear or be more readily cleaved, and these structures are then ruptured by contact stress and sliding. As a result, macroscopic removal rate and damage are governed jointly by the degree and spatial distribution of chemical activation and by the specific way in which mechanical loading acts on the weakened interfacial network. Within the current multiscale toolbox, DFT is best suited to identifying feasible reactions and adsorption states and to providing parameters for larger-scale models; classical MD clarifies mechanical contact boundaries and damage initiation; and ReaxFF-MD captures the full atomistic sequence from chemical softening and bridge-bond formation to stress-assisted rupture and removal.

Taken together, these modeling levels can be connected through physically meaningful information handoffs. At the atomistic level, DFT-derived reaction energetics, adsorption states, and interfacial chemical information can be used to parameterize reactive force fields or provide reaction-related boundary conditions for larger-scale models [107]. At the mesoscale, contact and transport models translate local interfacial behavior into quantities such as real contact area, local pressure, slurry-film characteristics, and local removal functions; for example, Seok et al. propagated single-asperity contact relations into statistical contact-pressure and material-removal calculations [79]. These physically meaningful quantities can further support data-driven modeling. Pad-surface descriptors have been incorporated directly into MRR prediction [47], while Yu et al. coupled physics-based wear information with machine learning to infer asperity radius and density before returning these quantities to a mechanistic MRR model [124]. At the process level, spatially resolved predictions of MRR and within-wafer non-uniformity can provide feedback for R2R control, including adjustment of zone-specific pressure setpoints [48]. Such handoffs provide a concrete pathway from atomistic mechanisms and mesoscale interfacial behavior to wafer-scale prediction, machine learning, and process control, although a fully standardized end-to-end framework has not yet been established.

## 5. Machine Learning in CMP

The CMP process in semiconductor manufacturing is characterized by complex interactions among chemistry, mechanics, transport, equipment condition, and consumable aging. Under such circumstances, purely physics-based models often struggle to maintain both accuracy and transferability across materials, tools, and operating conditions. Machine learning offers an attractive alternative and complement because it can process high-dimensional data, capture nonlinear dependencies, and exploit large volumes of process signals that are difficult to formalize analytically. Recent years have therefore seen rapid growth in the application of machine learning to CMP, particularly for process prediction, monitoring, virtual metrology, and intelligent control.

The diverse manufacturing challenges in CMP have driven the adoption of different machine-learning strategies. Regression-based methods are primarily used to predict continuous process outcomes, such as material removal rate and surface roughness, from process parameters and sensor information [125]. Classification approaches address discrete decision tasks, such as distinguishing defect types or process states, and have been applied to CMP-related surface-condition recognition [126]. Unsupervised approaches, including clustering, can group related products, wafers, or process conditions according to latent similarities and thereby support process-state analysis and model construction [127]. Anomaly-detection methods are used to identify deviations from normal process behavior and have been applied to real-time CMP monitoring using sensor signals [128]. Together, these learning paradigms support the major intelligent-CMP tasks of process prediction, state recognition, abnormal-condition detection, and subsequent decision support. The following sections discuss their applications in prediction, monitoring, virtual metrology, and process control in greater detail.

### 5.1. Machine Learning for Predicting Key CMP Metrics

Among all CMP targets, material removal rate and surface quality remain the most central because they directly influence throughput, uniformity, and yield. Yet both are jointly determined by slurry chemistry, load and speed, pad condition, pattern density, and tool drift. Traditional analytical models derived from the Preston equation or simplified contact mechanics can only accommodate a limited number of inputs and are often insensitive to evolving equipment structure, pattern effects, and noise. As machine learning has matured in manufacturing, CMP has increasingly been recast as a high-dimensional nonlinear mapping problem in which data-driven models refine—or, in some cases, substitute for—traditional empirical relationships.

#### 5.1.1. Prediction of Material Removal Rate

Early MRR prediction studies typically used process parameters as inputs and MRR as a single output in supervised regression. Representative examples include deep belief networks for learning nonlinear mappings and generating probabilistic predictions [129], as well as neural-network-based models trained on copper-CMP experiments to capture the complicated relationship between polishing parameters and removal rate [130]. These studies showed that data-driven models can accommodate much richer parameter interactions than conventional formula-based approaches.

With expanding datasets and computing resources, researchers began introducing more expressive architectures to improve generalization. Zhang et al. employed a residual convolutional neural network (ResCNN) for MRR prediction and achieved an MSE of 6.72 and an R^2^ of 0.9923 on the PHM2016 benchmark dataset, outperforming the compared CNN and conventional regression models [131]. Liu et al. developed a fusion network that encodes domain knowledge as shallow features while learning higher-order interactions through a deep architecture, thereby combining interpretability with strong representation power in a single framework [132]. The appeal of such deep learning models lies in their ability to automatically extract complex patterns from high-dimensional and nonlinear process variables and monitoring signals. At the same time, CMP datasets are often limited in size, noisy, and subject to distribution drift caused by consumable wear and equipment-state changes, so models optimized only for training accuracy may degrade substantially after a pad change, tool switch, or lot transition. Future work should therefore prioritize feature engineering and validation strategies that explicitly address drift-prone state variables. For surface-related inputs, it is also important to identify the observation scales that are most informative for the specific prediction target.

To improve practical utility, recent research has increasingly combined multiple information sources. One direction embeds process knowledge or measurable intermediate variables as physical constraints; another explicitly encodes the higher-order relationships among equipment components and multisource process signals. Yu et al. combined contact-mechanics reasoning with machine learning by first predicting pad-surface topography descriptors, such as asperity radius and density, and then coupling these outputs back into a physics-based MRR model [124]. Under small-sample conditions, Zhou et al. combined response surface methodology with neural networks to optimize and predict copper-CMP behavior using only a limited set of experiments [133]. To better represent interactions within the equipment and process state, Xia et al. introduced a hypergraph convolutional network for MRR prediction [134], and Huang et al. later extended this concept into a multimodal attention-based dynamic hypergraph contrastive-learning framework that remained robust even under limited data availability [46]. Wei et al. focused on hidden variables such as pad morphology by extracting latent representations through a conditional probabilistic autoencoder and then using stacking-based ensemble learning for MRR prediction [135]. Jeong et al. instead quantified pad-surface topography directly and showed that deep ensemble learning could substantially reduce prediction error across broader operating conditions than models using only a single pad descriptor [47]. These studies collectively suggest that durable MRR prediction depends less on blindly deepening networks than on systematically including the drift-sensitive state variables that govern contact and reaction.

#### 5.1.2. Surface Roughness and Quality Prediction

When the prediction target expands from MRR to surface roughness and other surface-quality metrics, the modeling problem becomes more complex [99], because these outcomes are jointly influenced by chemical, mechanical, transport, and material-response processes whose dominant mechanisms vary across material systems. For SiC CMP, data-driven approaches have become a practical tool for handling this complexity. For SiC CMP, BP neural networks have been used to learn nonlinear relationships between process conditions and polishing outcomes. Deng et al. combined a BP neural network with a modified Preston equation to describe single-crystal SiC CMP and successfully captured the nonlinear dependence of both MRR and roughness on pressure, speed, and slurry chemistry [125]. This hybrid strategy offered a useful quantitative framework for understanding the synergy and competition between chemical oxidation and mechanical removal during process-window optimization.

However, many surface-quality models remain black boxes: they may predict the outcome accurately but do not clearly explain the role or physical contribution of the most influential factors. To improve interpretability, Geng et al. compared RBF, BP, and CNN models for electro-Fenton CMP of single-crystal SiC and combined them with SHAP analysis to rank the contributions of individual process variables to MRR and surface quality [70]. This direction is particularly important for industrial deployment, where model acceptance depends not only on prediction accuracy but also on the ability to support process decisions. At present, surface-quality prediction remains at an exploratory stage. Most available studies focus on specific materials or fixed process windows, and model stability across different scenarios is still insufficiently validated [125]. The field is nevertheless moving toward richer data inputs, including multimodal and time-dependent process signals [46], measured pad-surface topography descriptors [47], and real-time monitoring data for anomaly detection [128], in order to improve sensitivity to drift and process variation. In this broader view, the value of machine learning lies not only in reducing prediction error, but also in identifying the variables that truly control surface integrity and thus shortening the convergence time of process-window development.

### 5.2. Machine Learning for Monitoring and Process Control

#### 5.2.1. Monitoring and Defect Identification

**Figure 8:**
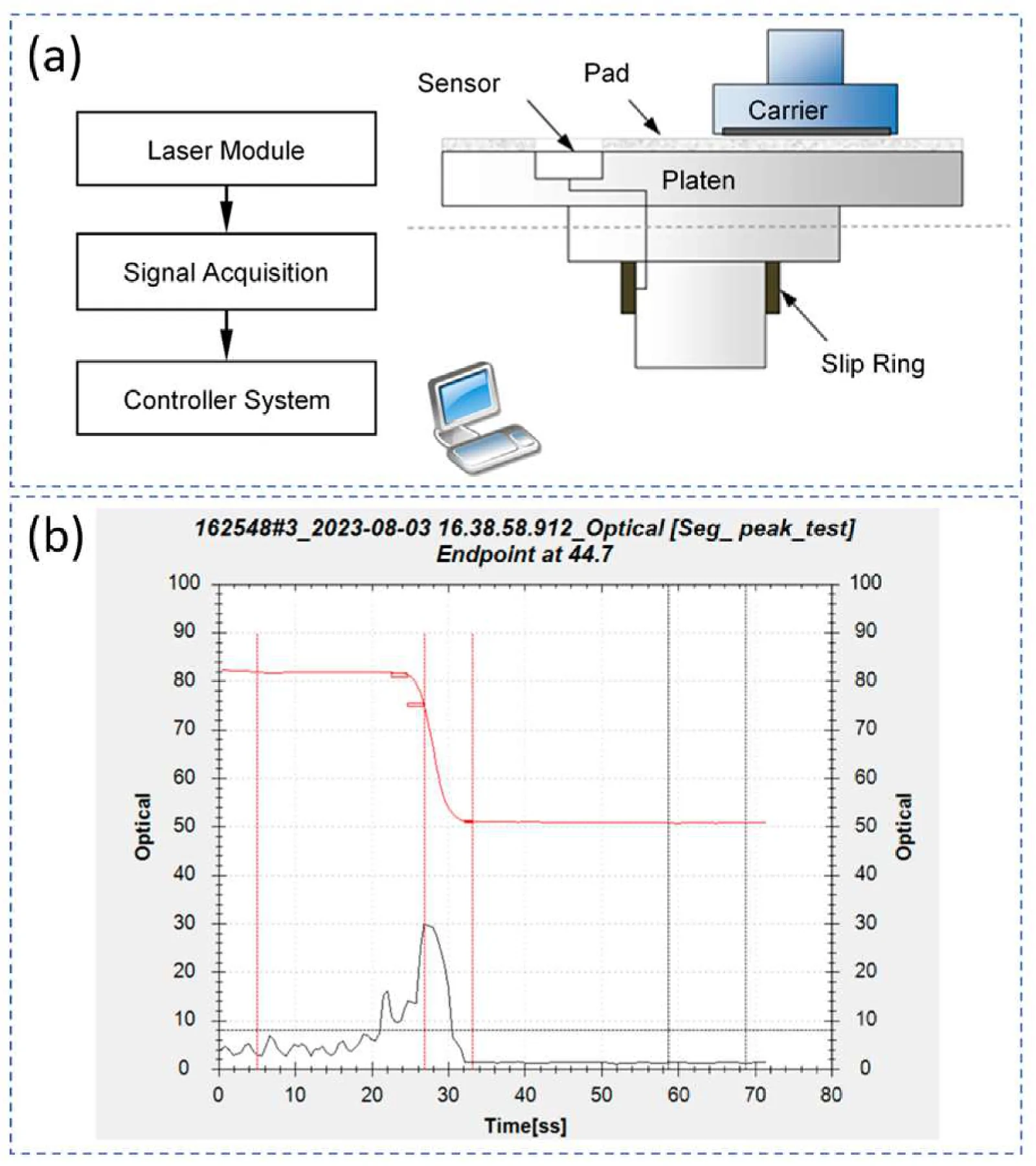
Representative in situ optical monitoring and endpoint detection in CMP: (**a**) schematic of the optical monitoring system integrated with the CMP tool and (**b**) evolution of the optical signal during polishing for endpoint identification. In panel (**b**), the red curve represents the mean optical-signal detection curve, whereas the gray curve represents the max–min (peak) curve of the optical signal profile. Adapted from Ref. [137] under the CC BY 4.0 license.

Because CMP involves strongly coupled chemical–mechanical interactions and evolving equipment states, process drift and abnormal events are common and must be detected promptly if stability is to be maintained. Traditional statistical process-control tools such as Shewhart, EWMA, and CUSUM charts are widely used in industry [136], but they are most effective when the monitored data are approximately unimodal and Gaussian. CMP data, however, are often non-normal or even multimodal, which limits the sensitivity and timeliness of conventional chart-based monitoring. Liu et al. addressed this challenge by introducing a real-time monitoring approach based on Dirichlet process Gaussian mixture models (DP-GMM), which can adaptively fit complex data distributions without prescribing the number of mixture components in advance [128]. Compared with EWMA-based monitoring, the proposed framework reduced the pad-wear detection delay to approximately 47 ms, compared with more than 140 ms for EWMA, while achieving an experimental F-score of 0.7923 [128], indicating that nonparametric Bayesian monitoring can significantly improve anomaly detection in CMP. A representative in situ optical monitoring system and its signal evolution during CMP are shown in Figure 8.

**Figure 9:**
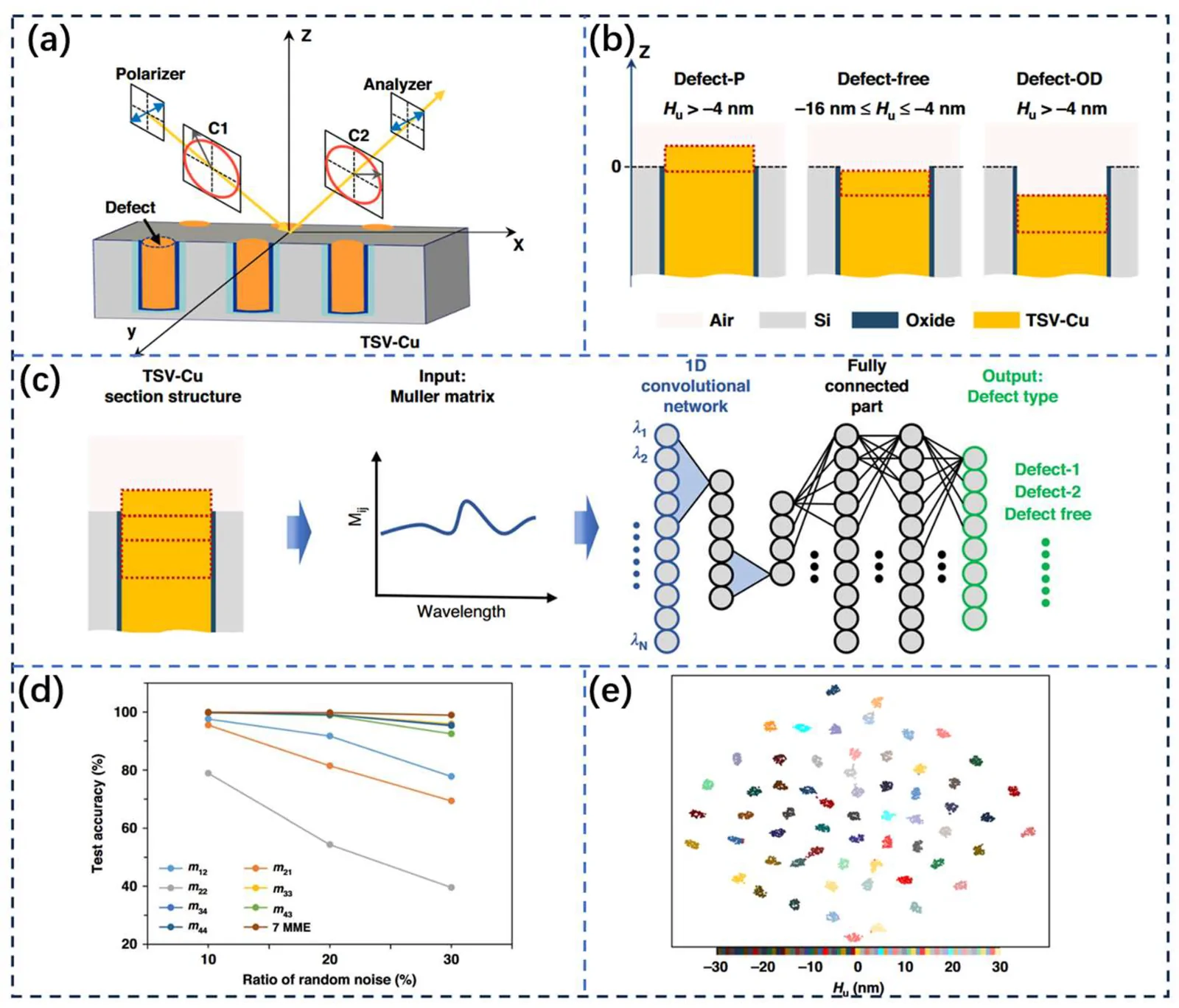
Nondestructive monitoring of TSV-Cu surface morphology using Mueller-matrix spectroscopic ellipsometry and convolutional neural networks, adapted from Ref. [126] under the CC BY 4.0 license: (**a**) optical simulation model of the TSV-Cu structure; (**b**) schematic of defect categories; (**c**) combined CNN and fully connected network for classification; (**d**) comparison of single- and multi-channel recognition accuracy under 10%, 20%, and 30% random noise; and (**e**) t-SNE visualization of the TSV feature space with 1 nm height resolution. The red dashed boxes in panels (**b**,**c**) indicate the TSV-Cu surface-height regions used to define the different defect states.

In addition to statistical monitoring of process variables, automatic detection of wafer-surface defects is a major concern in semiconductor manufacturing. Traditional inspection often relies on manual examination or machine-learning pipelines based on handcrafted features, which suffer from low efficiency and limited robustness when defect morphologies are complex. Saqlain et al. addressed this limitation by proposing a deep convolutional neural network for wafer defect identification on imbalanced datasets [138]. Their CNN-WDI model uses raw wafer-defect images as input, extracts high-dimensional features through convolutional layers, and combines batch normalization and dropout-based regularization to mitigate class imbalance. On a public nine-class wafer defect dataset, the method achieved an average classification accuracy of 96.2%, more than six percentage points higher than earlier baselines, and was especially effective for minority classes such as local scratches and random defects. Beyond classification, target-detection frameworks have also been introduced for rapid localization of manufacturing defects. Xing and Jia integrated a convolutional network classifier with YOLOv3 to enable real-time defect localization at approximately 23 frames per second while preserving high positional accuracy [139]. A representative deep-learning-assisted Mueller-matrix monitoring framework for TSV-Cu surface morphology is shown in Figure 9.

Recent work further suggests that CMP monitoring is moving from vision-only inspection toward multimodal sensing. Sun et al. used Mueller-matrix spectroscopic ellipsometry to obtain polarization-sensitive signals during TSV-Cu annealing and polishing, and then employed deep learning to fuse multiple Mueller-matrix elements for nondestructive monitoring of topography evolution [126]. This approach not only distinguished normal and defective wafer states with an accuracy exceeding 99%, but also achieved height sensitivity at the 1 nm level, allowing quantitative identification of polishing-induced dishing and annealing- or under-polishing-induced protrusions. Such results illustrate the promise of combining high-sensitivity sensing modalities with deep learning: statistical monitoring can warn of subtle distributional shifts, while image or optical-spectral analysis can identify and quantify the actual defects, together forming a richer basis for process intervention.

#### 5.2.2. Virtual Metrology

**Figure 10:**
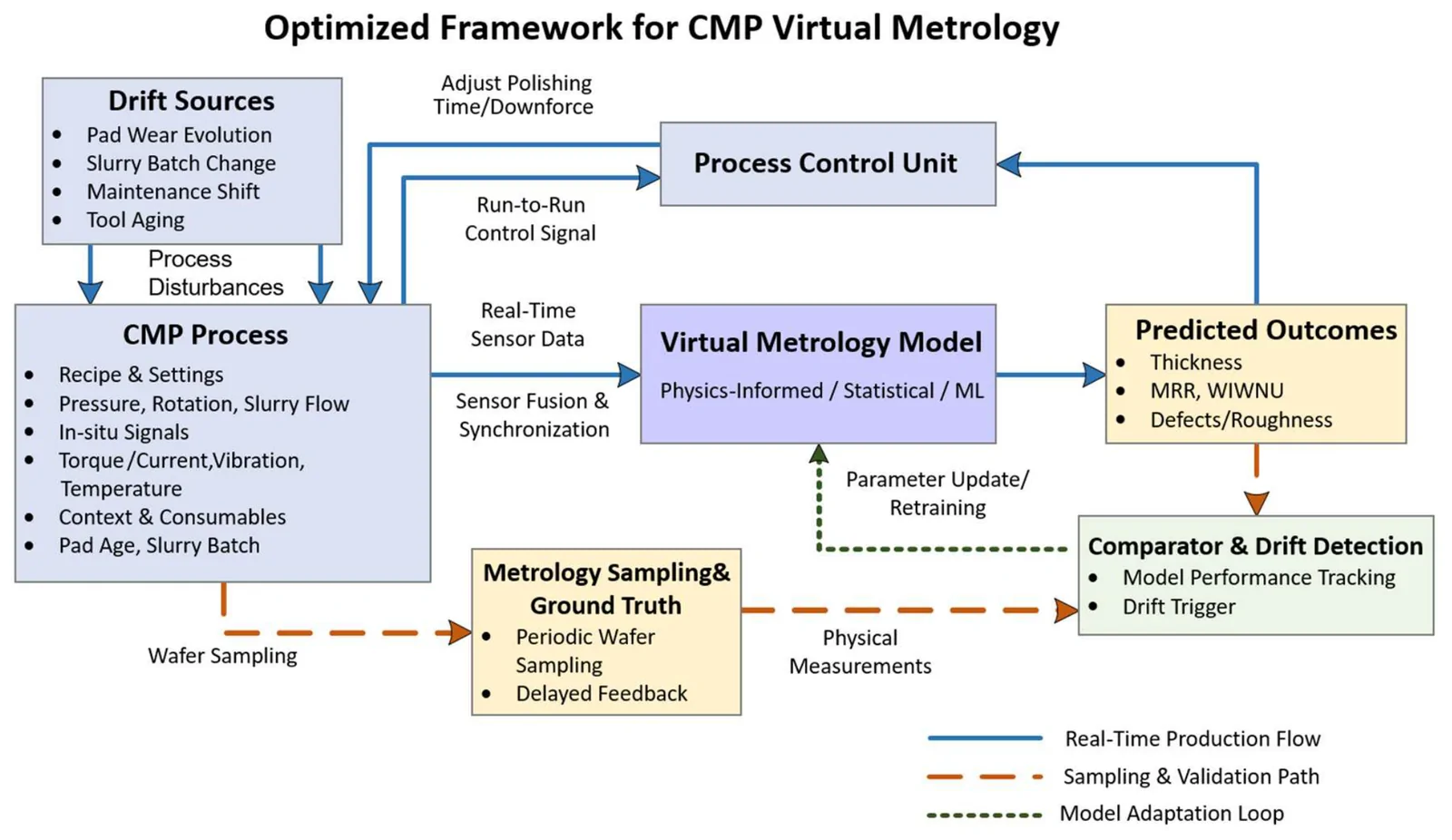
Optimized framework for CMP virtual metrology.

To reduce measurement cost and accelerate feedback, advanced process control in semiconductor manufacturing increasingly relies on virtual metrology (VM), in which process-state information is used to predict quality variables without adding physical metrology steps [140,141]. In CMP, nonlinear process drift is one of the main threats to VM accuracy. Lee and Kim proposed a recurrent feature-incorporated convolutional neural network (RFI-CNN) to address this issue [142]. Their dual-branch architecture combines CNN-based extraction of static features with RNN-based tracking of dynamic drift, and a two-stage training strategy enables the joint learning of spatial and temporal features. On factory CMP datasets, the model reduced thickness-variation fluctuation by 8.48% relative to the best baseline elastic-net model, demonstrating the practical potential of deep VM for stabilizing CMP operation under nonlinear disturbances. The resulting optimized framework for CMP virtual metrology is summarized in Figure 10.

The PHM2016 CMP dataset has become a common benchmark for evaluating VM algorithms. Using this dataset, Zhang et al. combined random-forest feature screening with a ResCNN model and showed that the residual architecture outperformed standard CNNs as well as traditional methods such as support vector regression and random forests [131]. Yet the same study also highlights a practical tradeoff: deep architectures can be computationally heavy and may not easily meet the millisecond-level latency requirements of online monitoring, while their dependence on high-quality labels becomes problematic in dynamic production settings. To mitigate these limitations, later work has explored model compression, multi-source data fusion, data augmentation, and adaptive updating. Huang et al.’s multimodal attention-based dynamic hypergraph contrastive-learning framework is representative of this trend, as it fuses heterogeneous sensor information and temporal attention to maintain robust prediction under limited training data [46].

Similarly, Liu et al. proposed a feature-fusion deep network for VM prediction of CMP removal rate by separating process variables into shallow features and deep features; the former can incorporate physical knowledge, whereas the latter are learned from data through deep neural networks [132]. The fused architecture outperformed both competition-winning and ensemble-based baseline models on the PHM2016 dataset. These studies suggest that improving VM accuracy is not simply a matter of using deeper models; rather, it requires careful integration of temporal correlation, process nonlinearity, multisource sensing, and physically meaningful feature design. In this sense, VM functions not merely as a surrogate for missing metrology, but as an enabling layer for more stable and responsive advanced process control in CMP.

#### 5.2.3. Run-to-Run Control and Reinforcement Learning

Run-to-run (R2R) control is a core strategy in semiconductor advanced process control. It adjusts the recipe for the next wafer on the basis of the result from the previous wafer so as to reduce deviation and maintain process stability. Classical R2R schemes are usually built on linear models such as exponentially weighted moving average (EWMA) control [143] and its dual-EWMA extensions [144]. These methods have been widely adopted in semiconductor manufacturing, but they require prior assumptions about disturbance form and manual tuning of control weights. When the actual disturbances are strongly nonlinear or time-varying—as they often are in CMP—fixed-parameter linear controllers become difficult to optimize [145]. Because CMP is nonlinear, time-varying, and influenced by multiple disturbance sources, researchers have increasingly turned to machine learning and intelligent control to enhance R2R performance.

Neural-network-based R2R control was one of the earliest improvements in this direction. In this framework, separate neural networks are used to represent the process model and the controller, and their weights are updated directly from input–output data without requiring an explicit analytical process model. Earlier studies showed that such neural-network R2R control can yield a more uniform wafer-thickness profile in CMP [146] and improve the optimization of polishing inputs [147]. Building on this concept, Kim et al. introduced a least-squares generative adversarial network (LS-GAN)-based R2R controller that bypasses an independent VM module and instead directly learns a control-oriented representation of the process [148]. By embedding a CNN within the architecture to extract drift features and training the generator to approximate the actual process distribution, the controller became less sensitive to hyperparameters such as window size and outperformed conventional neural-network R2R control under both drift and abrupt-change conditions. Bayesian optimization was further used to stabilize GAN training and improve convergence.

To further improve adaptability under complicated disturbances, reinforcement learning (RL) has recently been introduced into CMP control. RL can learn approximately optimal control policies through repeated interaction with the environment without requiring a precise process model. Ma and Pan modeled recipe adjustment in CMP as a Markov decision process and proposed a distributional reinforcement-learning controller, QUOTA-DDPG, for intelligent R2R tuning [149]. By defining states, actions, and rewards around disturbance compensation and target tracking, the method used distributional rather than point-estimate value learning to balance performance and stability. Simulation results showed clear advantages over classical R2R controllers in both disturbance rejection and target tracking. Ma and co-workers later proposed a DRL-dEWMA controller that uses deep reinforcement learning as an online tuning layer for a conventional dEWMA controller, thereby retaining the basic stability and interpretability of the classical architecture while improving its adaptability to time-varying disturbances.

More broadly, recent work in intelligent process control indicates that data-driven agents need to be integrated with prior knowledge and appropriate control constraints for application in complex manufacturing environments. In semiconductor process control, high-fidelity neural-network environments have been used to support deep-distributional soft actor-critic learning for nonlinear control tasks [150], suggesting a future direction in which learning-based optimization is integrated with established control structures to connect sensing, prediction, and actuation within an adaptive closed loop.

**Figure 11:**
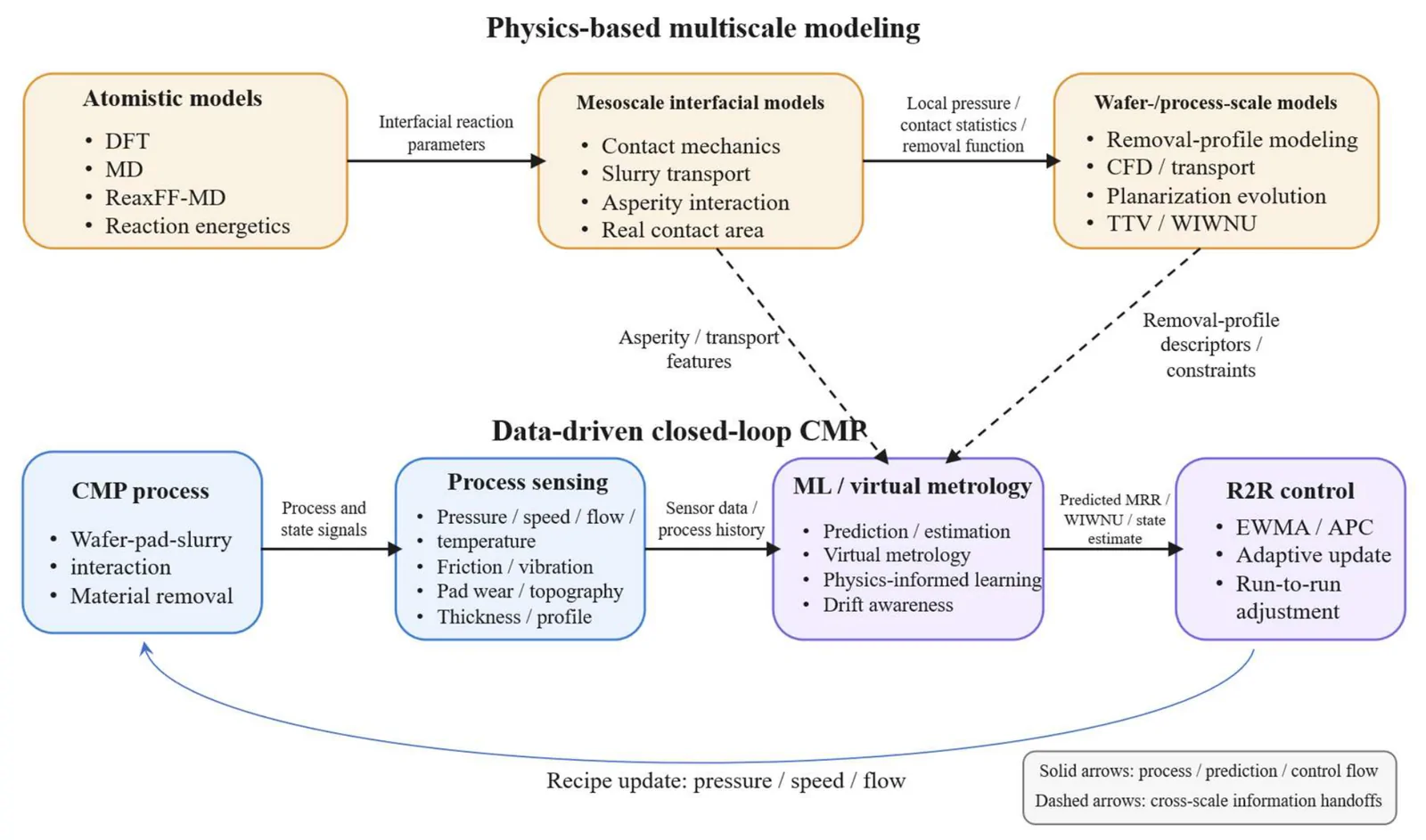
Cross-scale information flow linking physics-based multiscale modeling, process sensing, machine learning, and R2R control in CMP.

The preceding studies also indicate that intelligent CMP should not be viewed as a sequence of isolated modeling and control tasks. As summarized in Figure 11, atomistic and mesoscale models provide physically meaningful descriptors for wafer-scale prediction and data-driven modeling, while process sensing supplies real-time or run-level information to machine-learning and virtual-metrology models. Their outputs can subsequently support R2R recipe adjustment, forming a cross-scale information pathway from mechanistic understanding to process decision-making.

Recent quantitative results also suggest that several building blocks required for closed-loop CMP are approaching practical levels of performance. ResCNN-based MRR prediction achieved an MSE of 6.72 and an R^2^ of 0.9923 on the PHM2016 dataset [131], while a pad-state-aware model trained on 180 experimental samples reported an RMSE of 80.15 Å/min and an R^2^ of 0.9857 [47]. At the control level, neural-network-based R2R control reduced WIWNU from approximately 6–7% to 1–2% [146], and reinforcement-learning-based control maintained low tracking errors under multiple disturbance scenarios [149]. These results indicate that accurate prediction and adaptive control can already be achieved independently, providing a quantitative basis for their future integration with process sensing and virtual metrology. Nevertheless, end-to-end validation across tools, materials, and operating conditions remains an important open challenge.

To facilitate comparison across these modeling, prediction, monitoring, and control approaches, Table 2 summarizes representative multiscale and machine-learning studies in CMP, including their model scale, inputs and outputs, validation basis, reported performance, transferability, and computational considerations.

Beyond process-level prediction and control, machine learning provides opportunities for materials-informed CMP design [151]. By integrating material descriptors, experimental polishing data, and outputs from atomistic simulations, data-driven approaches can uncover relationships among abrasive/slurry characteristics, interfacial chemistry, and polishing performance [104,152]. Such materials-informatics strategies may enable accelerated screening of abrasives and additives, reducing reliance on extensive trial-and-error experiments and extending intelligent CMP from process optimization toward consumable design.

## 6. Sustainability in CMP

Most of the developments discussed above—whether in materials design [64,152], multiscale modeling [17,84], or machine learning [46,131]—have been motivated by the need to increase removal efficiency, improve planarization, and suppress defects. Although these efforts have significantly advanced process capability, they often pay less attention to the environmental costs of CMP. As semiconductor manufacturing flows grow longer and more complex, CMP has become one of the most water- and chemical-intensive operations in the fab [7]. It is estimated that CMP wastewater accounts for approximately 30–40% of total fab effluent, and that ultra-pure water consumption in CMP exceeds one-third of total fab water use [153,154]. These figures imply that future CMP technology cannot be judged solely by polishing performance; it must also be evaluated in terms of sustainability.

### 6.1. Process Optimization and Source Reduction

Improving resource efficiency and reducing consumption at the source are among the most direct ways to lower the environmental burden of CMP. Lee et al. provided a systematic review of sustainability pathways in CMP [155]. For wastewater rich in nano-abrasives such as CeO_2_ and SiO_2_, combinations of electrocoagulation [156], electrodialysis [157], chemical coagulation [158], and membrane filtration [159] have been used for deep treatment, enabling removal of suspended particles and part of the dissolved contaminants so that the treated water can satisfy stringent discharge or reuse requirements. These treatments also concentrate abrasives and metal ions into sludge or retentate streams, opening the door to resource recovery such as rare-metal extraction or abrasive regeneration [160]. CMP sludge itself can be reused as an additive in concrete [161], providing a practical example of industrial-waste valorization. On the process side, optimized slurry delivery systems—such as spray nozzles [162] and Araca-type slurry injection systems [163]—can reduce slurry usage while maintaining polishing performance. Mixed-abrasive strategies may also contribute indirectly to sustainability by improving MRR through synergy between large and small particles, thereby shortening polishing time and reducing energy and water consumption per wafer [64].

It is important to note, however, that end-of-pipe wastewater treatment and recycling do not eliminate pollution at the source and often introduce additional energy and operational costs. Sustainable CMP therefore also requires reformulating slurries and post-clean chemistries to reduce the use of persistent, highly complexing, or otherwise problematic compounds. Biodegradable, low-toxicity inhibitors and complexing agents should be prioritized whenever possible to lower the burden on wastewater systems. Because all interventions involve tradeoffs among removal performance, energy demand, consumable usage, and treatment difficulty, a full life-cycle assessment (LCA) framework is needed to place water, chemicals, energy, consumables, and disposal within a common evaluation boundary.

### 6.2. Multiscale Modeling and Digital Approaches to Sustainable CMP

Multiscale modeling and machine learning open new routes for sustainability-oriented optimization. Physics-based modeling can replace overly conservative empirical settings with computable process relationships, thereby reducing unnecessary consumption of water, slurry, energy, and consumables. By linking load, speed, temperature, flow behavior, and pad state to removal, uniformity, and defect-related indicators [80], such models can support more rational decisions on process time, slurry delivery, cleaning strategy, conditioning, and maintenance, and can reduce rework or premature consumable replacement caused by poorly controlled drift. Mechanism-based models may also help evaluate the residual behavior and treatability of slurry and post-clean chemicals, making it possible to favor components that are easier to degrade or recover.

Machine-learning-based virtual metrology and advanced process control can further reduce non-productive consumption. By predicting polishing outcomes from equipment sensor data, VM decreases reliance on physical metrology and monitor wafers, thereby saving wafer resources together with associated water and energy use [141]. Intelligent endpoint detection based on frictional or optical signals can also prevent over-polishing and its attendant waste of slurry, electricity, and tool time. At an earlier stage of process development, machine learning can reduce trial-and-error experimentation in the search for greener consumables. Venkataswamy et al. argued that screening environmentally friendlier additives by conventional experiments alone is inefficient and chemical-intensive; by combining computational chemistry with machine learning, one can rapidly estimate adsorption energies and corrosion-inhibition tendencies for biomolecules such as amino acids on specific metal surfaces, greatly narrowing the experimental search space [164]. More broadly, this represents a materials-informatics framework for sustainable CMP development. Future efforts could integrate molecular descriptors, simulation-derived properties, experimental slurry databases, and environmental metrics to enable multi-objective screening of consumables considering removal efficiency, surface quality, toxicity, recyclability, and resource consumption. Such an approach could help balance traditional CMP objectives, such as removal efficiency and surface quality, with emerging sustainability requirements during early-stage consumable development. Comparable descriptor-based screening strategies are already established in other materials domains, where machine-learning and materials-informatics approaches have been used to relate structural descriptors to the physical properties of nanostructured materials [165]; transferring such methodologies to CMP would, however, require CMP-specific descriptor sets and sufficiently large polishing datasets. Ultimately, sustainable CMP should be viewed as a system-level objective: efficient tools reduce resource demand, recyclable and benign chemistries lower environmental burden, and intelligent digital methods coordinate the entire process toward green manufacturing.

## 7. Conclusions and Future Perspectives

CMP research is increasingly moving beyond isolated improvements toward the coordinated development of materials, process technologies, mechanistic modeling, data-driven intelligence, and sustainability. Advanced abrasives, engineered slurry systems, and assisted CMP strategies can improve removal efficiency, surface integrity, and the process window, particularly for hard or chemically inert materials. However, their practical deployment is still constrained by synthesis complexity, batch consistency, consumable stability, cost, and the additional equipment and field-control requirements introduced by assisted processes. Sustainability-oriented approaches such as source reduction, recycling, and greener chemistries can reduce environmental burden, but their benefits must be balanced against treatment cost, energy use, and process performance. The application scope of CMP is also expanding beyond conventional semiconductor substrates. For example, CMP has recently been investigated as a surface-treatment route for improving the corrosion resistance of carbon steel in acidic media, where polishing-induced changes in surface roughness and wettability were associated with enhanced corrosion resistance [166]. Such extensions broaden the functional objectives of CMP and further expand the criteria by which consumables and process strategies should be evaluated.

Multiscale physical modeling provides interpretable descriptions of CMP from wafer-scale planarization to mesoscale contact and transport and atomistic reaction mechanisms, but its predictive transferability remains limited by calibration requirements and incomplete cross-scale coupling. Machine learning complements these models through nonlinear prediction, process monitoring, virtual metrology, and intelligent control, yet its industrial reliability is still affected by data quality, process drift, domain shift, limited transferability, and insufficient uncertainty quantification. Taken together, materials and process innovations provide direct performance gains, physics-based models offer mechanistic interpretability, and machine-learning methods provide flexible prediction and adaptation; however, their practical limitations differ, ranging from manufacturability and process complexity to cross-scale transferability, data quality, and generalization. These complementary strengths and limitations indicate that no single approach is sufficient for next-generation CMP.

Future CMP research should therefore focus on reliable cross-scale information transfer, physics-informed and uncertainty-aware learning, multimodal sensing, and closed-loop multi-objective optimization. A next-generation CMP ecosystem should connect consumable design, mechanistic modeling, in situ sensing, virtual metrology, and intelligent control, with sustainability metrics incorporated directly into process decisions. In such a framework, physical models can provide interpretable priors, data-driven methods can adapt to evolving process conditions, and lifecycle considerations can guide the simultaneous optimization of planarity, damage, yield, resource use, and environmental impact.

## Figures and Tables

**Table 1:** Quantitative comparison of representative experimental materials and process strategies for CMP.

Material/Substrate	Strategy and Key Conditions	MRR/Processing Rate	Roughness/Damage/Selectivity	Key Limitation/Practical Consideration	Ref.
**A. Abrasive and slurry design**					
ZrO_2_ ceramic	Peanut- and heart-shaped SiO_2_ abrasives prepared by KH550-induced morphology control	0.31 μm/h	Ra = 1.9 nm	Requires controlled synthesis of non-spherical SiO_2_ abrasives	[24]
Sapphire	PS@CeO_2_/DND ternary core–shell abrasives; pad speed 120–150 r/min; load ≈ 3 kg	1.4–1.7 μm/h	Ra = 0.52 nm	Requires preparation and assembly of multi-component core–shell abrasives	[29]
SiO_2_ film	La-doped porous self-assembled CeO_2_ abrasives	338 ± 7.54 nm/min; 35% higher than undoped porous CeO_2_	Ra = 0.201 ± 0.010 nm	Requires simultaneous control of rare-earth doping and porous abrasive structure	[49]
Co/Ti interconnect	SiO_2_–H_2_O_2_ slurry with potassium citrate; pH 9, 3 wt% SiO_2_, 1 wt% H_2_O_2_, 10 mM potassium citrate	Co: 18.05 nm/min; Ti: 37.35 nm/min	Ti Ra = 0.14 nm; Co:Ti removal selectivity = 1:2	Slurry chemistry must balance Ti removal and Co/Ti selectivity	[27]
Zinc germanium phosphide (ZGP)	Green mixed-abrasive slurry containing SiO_2_, CeO_2_, and Na_2_CO_3_; oxidant- and etchant-free	11.9 μm/h	Ra = 0.084 nm; damage-layer thickness = 3.1 nm	Performance relies on a material-specific mixed-abrasive chemistry	[61]
4H-SiC	Al_2_O_3_@MIL-100(Fe) composite abrasive in photocatalytic-assisted CMP; pH 6, 3 wt% persulfate, 22 wt% MIL-100(Fe), UV irradiation	417 nm/h	Sa = 2.17 nm	Requires MOF-composite synthesis together with persulfate and UV activation	[30]
**B. Assisted and advanced process strategies**					
4H-SiC	Photoelectrochemically combined mechanical polishing (PECMP); 6.5 wt% PS@CeO_2_ abrasives, CeO_2_–TiO_2_ photocatalyst, UV irradiation and anodic bias	1.109 μm/h	Ra = 0.738 nm; verification test: Ra = 0.113 nm with a scratch-free surface and less residual oxide	Requires coupled UV irradiation, anodic bias, and photocatalytic components	[28]
Single-crystal SiC	Electro-Fenton CMP using H_2_O_2_/Fe_3_O_4_; acidic environment (~pH 3); voltage and mechanical parameters jointly optimized	1458 nm/h	Ra = 0.356 nm	Requires coordinated optimization of electrochemical and mechanical conditions	[20]
Single-crystal diamond	Green photocatalytic CMP using H_2_O_2_, diamond abrasives, and PB/TiO_2_ catalyst under UV irradiation	1168 nm/h	Sa = 0.079 nm; damage-layer thickness = 0.66 nm	Requires UV irradiation and photocatalyst control	[65]
Sapphire	Oxygen gas-assisted CMP (GA-CMP); dissolved O_2_ concentration = 40 ppm	MRR increased by 23.02% relative to conventional CMP	Ra = 0.124 nm; 57.3% reduction relative to conventional CMP	Requires controlled enrichment and regulation of dissolved oxygen	[68]
Lu_2_O_3_	Plasma-assisted etching combined with low-pressure polishing; plasma modification by hydroxyl species and 25% H_2_SO_4_ etching	Plasma modification rate = 136 nm/h	Sa = 0.14 nm; atomically flat and SSD-free surface; total processing time reduced by ~50%	Requires integration of plasma modification, chemical etching, and low-pressure polishing	[73]
4H-SiC	Friction-induced chemical reaction polishing using pure Fe; solid-state reaction under relatively low speed, pressure, and temperature	375 nm/min	Ra = 2.2 nm; near-damage-free surface/subsurface; demonstrated on a 4-inch wafer	Requires direct reactive contact between pure Fe and SiC rather than a conventional slurry-based CMP configuration	[74]

**Table 2:** Synthesis of representative multiscale and machine-learning approaches for CMP.

Study	Scale/Method	Inputs/Outputs	Dataset/Validation	Reported Performance	Transferability/Computational Cost
A. Physics-based and multiscale modeling					
Zhao et al. [81]	Wafer scale; CFD–Preston model	Inputs: initial surface topography, pressure distribution, relative velocity, Preston coefficient; output: wafer flatness (TTV)	GaAs polishing experiments + numerical simulation; five repeats per setting	TTV: 50 → 36.82 μm; predicted TTV = 36.30 μm at $w_{h}=w_{d}=40$ rpm	Requires $k_{p}$ recalibration for new processes; FEM-based computation
Xu et al. [82]	Chip–feature scale; two-scale mechanistic model	Inputs: global/local pressure, slurry chemistry; outputs: surface-profile evolution, W dishing	Patterned-W CMP experiments	Simulated W dishing nearly quantitatively consistent with experiments across line width and CTAB concentration	Patterned-W-specific; full-chip contact calculation is costly
Wang et al. [89]	Mesoscale; contact-mechanics model	Inputs: contact ratio, spot size/spacing, chemical/mechanical factors; output: MRR	Experiments on BK7, fused silica, and monocrystalline Si	Predicted MRR showed good agreement with experiments; MRR a/MRR b ≈ 1.3 for BK7 and ≈1.0 for Si	Cross-material validation; material/pad parameters require recalibration
Zhou et al. [93]	Mesoscale; DPM/DDPM transport model	Inputs: slurry concentration, abrasive size, pad/wafer speed; outputs: particle transport, removal behavior	CFD + AFM/polishing experiments	MRR: 2.29–10.90 × 10^−9^ nm/s; AFM-validated	DPM limited to dilute flows; DDPM has higher computational cost
Yang et al. [99]	Microscale; topography-evolution model	Inputs: reaction layer, abrasive removal, pad contact, pH, particle size; outputs: Ra, PSD	Single-crystal Si polishing experiments	Ra error: 31.6–40.6%; PSD error: up to ≈50%	Requires re-identification of chemical/mechanical/contact parameters
Dang et al. [105]	Atomistic; DFT + mechanochemical analysis	Inputs: surface chemistry, EDA adsorption; outputs: reaction barriers, MRR/selectivity, Sa	DFT + etching/XPS/AFM/CMP experiments	Poly-Si MRR > 1000 nm/min; Sa ≈ 0.3 nm; high selectivity	Chemistry/interface-specific; DFT computationally intensive
Li et al. [114]	Atomistic; ReaxFF-MD	Inputs: pH, pressure, slip velocity; outputs: atomic-removal behavior, MRR, Sq	ReaxFF-MD + polishing experiments	Min. Sq = 0.126 ± 0.002 nm (MRR = 251.47 ± 0.99 nm/min); max. MRR = 593.56 ± 0.95 nm/min (Sq = 0.276 ± 0.004 nm)	Force-field/material-dependent; ReaxFF-MD computationally intensive
B. Machine-learning-based prediction, monitoring, and control					
Yu et al. [124]	Hybrid physics–ML; random forest	Inputs: CMP process variables and pad/dresser usage; output: MRR	PHM2016;	Test RMSE = 15.770 nm/min, RE = 0.114; validation RMSE = 16.908 nm/min, RE = 0.120	Limited cross-stage transferability: Stage A and B required separately trained models
Zhang et al. [131]	Process scale; RF + ResCNN	Inputs: six RF-selected usage features + chamber ID; output: MRR	PHM2016;	MSE = 6.72; R^2^ = 0.9923	Scenario-specific; separate models for stage/chamber scenarios
Jeong et al. [47]	Pad-state-aware ML; deep ensemble	Inputs: polishing pressure + 15 pad-surface topography descriptors; output: MRR	180 experimental samples	RMSE = 80.15 Å/min; MAPE = 3.283%; R^2^ = 0.9857	Pad/material-specific features; cross-tool/material transfer untested
Geng et al. [70]	Process scale; RBF/BP/CNN + SHAP	Inputs: electro-Fenton process parameters; outputs: MRR, Ra	23 training + 4 independent test experiments; 5-fold CV	BP test RMSE = 101.19 nm/h for MRR; 0.03 nm for Ra	Small SiC-specific dataset; cross-tool/material transfer untested
Liu et al. [128]	Process monitoring; DP-GMM	Input: wireless vibration signals; outputs: anomaly detection and process-state identification	Numerical simulations + experimental CMP testbed	Pad-wear detection ≈47 ms vs. >140 ms for EWMA; experimental RHDP F-score = 0.7923	Sensor/test-bed-dependent; machine transfer requires signal adaptation
Sun et al. [126]	Multichannel monitoring; ellipsometry + deep learning	Inputs: Mueller-matrix signals; outputs: defect class, feature height	Simulation-augmented data; 70/30 train/test split	Classification accuracy = 99.94%; height resolution = 1 nm	TSV geometry/optical-model-dependent; new structures require retraining
Lee and Kim [142]	Virtual metrology; RFI-CNN	Inputs: static and time-dependent CMP variables; output: wafer-edge thickness	Factory CMP process data	Process variability (SD) reduced by 8.48% vs. elastic net	Partial cross-tool transfer; C2 required 5000-wafer pretraining; online fine-tuning
Yi et al. [146]	R2R control; neural-network models	Inputs: RR/uniformity profile, air-zone pressures, platen height; outputs: next-run settings and controlled RR/uniformity profile	32-run DOE + 500-wafer R2R simulation	WIWNU = 1–2% vs. 6–7% uncontrolled; controlled RR ≈ 3550 Å/min vs. 100–200 Å/min uncontrolled drift	Lam linear-CMP-specific; LM ≈ 9 ms/iteration; BP ≈ 113 ms/iteration
Ma and Pan [149]	R2R control; distributional RL	Inputs/state: previous MRR, tracking error, previous downforce/velocity; outputs: next-run downforce/velocity; controlled MRR	50 × 600-run simulation; five disturbance scenarios	MRR RMSE = 0.04727 (DT), 0.21146 (shift), 0.06192 (drift), 0.10705 (IMA), 0.11319 (ARIMA)	Simulation-only MISO validation; MIMO/dynamic transfer untested; CPU time = 988.95 s

## Data Availability

No new data were created or analyzed in this study. Data sharing is not applicable to this article.
